# Circulating Adipocytokines and Insulin Like-Growth Factors and Their Modulation in Obesity-Associated Endometrial Cancer

**DOI:** 10.3390/cancers16030531

**Published:** 2024-01-26

**Authors:** Irene Ray, Carla S. Möller-Levet, Agnieszka Michael, Simon Butler-Manuel, Jayanta Chatterjee, Anil Tailor, Patricia E. Ellis, Lisiane B. Meira

**Affiliations:** 1 Department of Clinical and Experimental Medicine, University of Surrey, Daphne Jackson Road, Guildford GU2 7WG, UK; 2 Academic Department of Gynaecological Oncology, Royal Surrey NHS Foundation Trust, Egerton Road, Guildford GU2 7XX, UK; 3 Bioinformatics Core Facility, University of Surrey, Daphne Jackson Road, Guildford GU2 7WG, UK; 4 Department of Oncology, Royal Surrey NHS Foundation Trust, Egerton Road, Guildford GU2 7XX, UK; 5 Department of Life Sciences, Brunel University London, Kingston Lane Uxbridge, Middlesex, Uxbridge UB8 3PH, UK

**Keywords:** adipocytokines, insulin-like growth factor, endometrial cancer risk, obesity, body mass index

## Abstract

**Simple Summary:**

The rise in worldwide uterine cancer is tied to increasing obesity, influencing substances such as adipocytokines and insulin-like growth factors (IGFs) in the body, promoting cancer mainly through inflammation. Our main objective was to evaluate the levels of adiponectin, leptin, TNFα, IL6, IGFs 1 and 2 in endometrial cancer patients compared to control patients and to examine their relationship with obesity. Additionally, we aimed to explore the correlation between these markers and tumour characteristics. We also conducted a reassessment of the markers 6 months post-surgery to investigate the impact of treatment on these markers. Given the absence of established biomarkers for endometrial cancer, studying these markers and their variations post-surgery may provide valuable prognostic insights.

**Abstract:**

The rising global incidence of uterine cancer is linked to the escalating prevalence of obesity. Obesity results in alterations in adipocytokines and IGFs, driving cancer progression via inflammation, increased cell proliferation, and apoptosis inhibition, although the precise mechanisms are still unclear. This study examined a set of six markers, namely, adiponectin, leptin, IL6, TNFα, IGF1, and IGF2 and compared them between fifty age-matched endometrial cancer patients (study group) and non-cancer patients with benign gynaecological conditions (control group). We also assessed the relationship of these markers with obesity and explored the correlation between these markers and various tumour characteristics. In the cancer population, these markers were also assessed 24 h and 6 months post-surgery. Remarkably, low adiponectin levels were associated with a 35.8% increase in endometrial cancer risk. Interestingly, compared to control subjects where IGF levels decreased after menopause, post-menopausal women in the study group showed elevated IGF1 and IGF2 levels, suggesting a potential influence of endometrial cancer on the IGF system, particularly after menopause. Lastly, it is noteworthy that a discernible inverse relationship trend was observed in the levels of adipocytokines and IGFs 6 months post-surgery. This indicates that treatment for endometrial cancer may have a differential impact on adipocytokines and IGFs, potentially holding clinical significance that merits further investigation.

## 1. Introduction

Uterine cancer is the most prevalent cancer of the female reproductive system in the UK, affecting 1 in 36 women (3%), with 9500 cases diagnosed annually [1]. Obesity is a significant risk factor, contributing to 34% of cases and a 16% higher risk per 5 kg of weight gain in adulthood [2]. This risk affects both pre- and post-menopausal women and is associated with poorer outcomes in obese patients [3].

**Figure 1:**
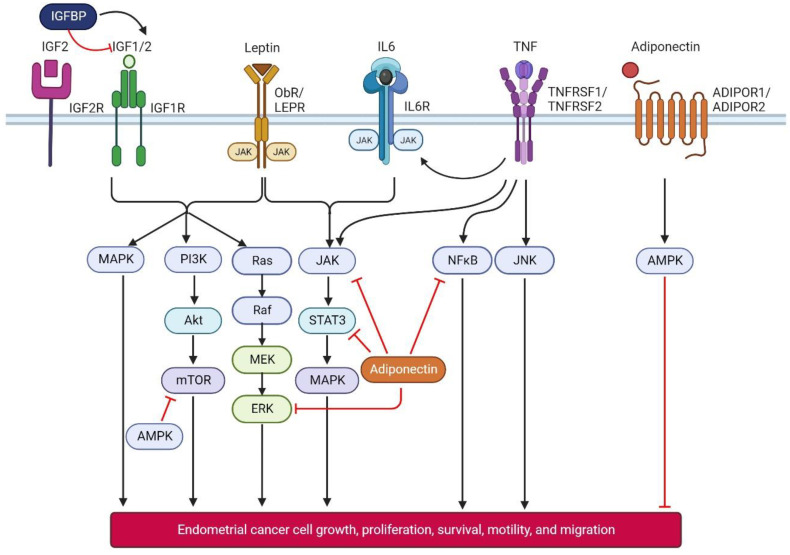
Schematic diagram showing interactions of major adipocytokines and IGFs and their downstream signalling pathways. The black arrows indicate a stimulatory effect, and the red blocked arrows indicate an inhibitory effect. AMPK, AMP-activated protein kinase; ERK1/2—extracellular regulated kinase 1 or 2; JAK—janus kinase; JNK—c-Jun NH2-terminal kinase; MAPK—mitogen-activated protein kinase; mTOR—mammalian target of rapamycin; MEK—MAPK/ERK Kinase; NF-κB—nuclear factor-κB; P13K/Akt—phosphatidylinositol 3-kinase/protein kinase B; Ras—Rat Sarcoma protein; Raf—Rapidly Accelerated Fibrosarcoma protein; STAT3—signal transducer and activator of transcription. Figure created with BioRender.com (accessed on 12 November 2023). Data source: [45,46,47,48,49].

The inflammation resulting from excess adipose tissue is a key contributor to endometrial cancer development [4]. Adipokines and inflammatory cytokines produced by adipocytes and macrophages, such as adiponectin, leptin, TNFα, and IL6, can promote tumour growth by cell proliferation, migration, and neo-angiogenesis (Figure 1) [5]. Dysregulated adipocytokine signalling pathways create an inflammatory microenvironment that inhibits cell apoptosis and induces cell proliferation, increasing the risk of endometrial cancer.

**Figure 2:**
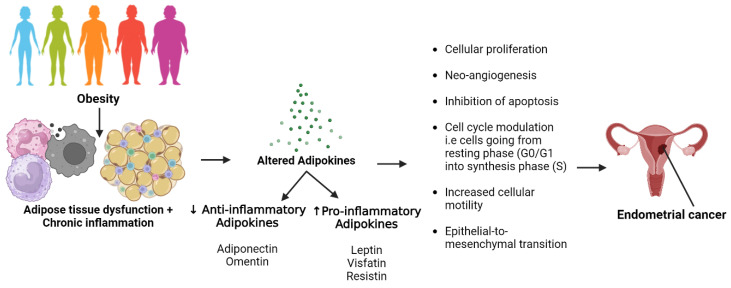
Role of adipokines in endometrial development. Figure created using Biorender.com (accessed on 11 January 2024). Data source: [53,54,55,56].

Observational studies have associated the development of diseases such as breast cancer with adipose tissue, particularly emphasising the role of adipokines secreted within its microenvironment. The repertoire of these adipokines is continuously expanding, encompassing examples such as leptin, visfatin, resistin, osteopontin, and others [6,7]. Among these adipokines, adiponectin and leptin are the best studied. Adiponectin, with its insulin-sensitising and anti-inflammatory properties, plays a crucial role in reducing risk of various cancers such as breast cancer [8,9], ovarian cancer [10], cervical cancer [11], endometrial cancer [12], colorectal cancer [13], and prostate cancer [14]. In contrast, leptin was primarily linked to food intake and energy expenditure [6] and has been associated with increased cancer risk due to its involvement in proinflammatory immune responses, angiogenesis, and cell proliferation in colorectal cancer [15], lung cancer [16], glioma [17], breast cancer [18], and endometrial cancer [4,19]. The role of adipokines in endometrial cancer development is illustrated in Figure 2. The adiponectin/leptin ratio (A/L ratio) is thought to serve as an indicator of adipose tissue dysfunction, and an elevated ratio is linked to lower risks of atherosclerosis and certain cancers, like breast cancer [20,21].

Cytokines like IL6 and TNFα are secreted in the tumour microenvironment. Cytokines can either support anti-tumoural responses or contribute to cancer development and metastasis, depending on the balance between pro- and anti-inflammatory cytokines [22,23]. The relationship between cytokines and endometrial cancer is debated, as some studies indicate a connection with elevated levels, while others find no correlation [24,25].

Obesity has also been linked to dysregulation of IGF levels, through either increased IGF production or down-regulation of IGF binding proteins due to obesity-induced prolonged insulin resistance and hyper-insulinemia (“insulin-IGF hypothesis”) [26]. This link has been demonstrated especially for oestrogen-dependent type I endometrial cancer [27]. The IGFs are powerful growth factors that contribute to tumour growth and proliferation by promoting cell proliferation and inhibiting apoptosis [28]. IGF1 is associated with an increased risk of colorectal [29], prostate [30], breast [31], and lung [32] cancers, while IGF2 is implicated in colorectal cancer [33,34], liver cancer [35], and prostate cancer [30]. Both IGFs have been reported to be associated with endometrial cancer risk, though a definite risk relationship has not been conclusively established [36]. Adipocytokines and IGFs have also been linked with the progression of endometrial cancer, but the mechanisms remain unclear [37]. They have been linked with prognostic factors of endometrial cancer such as grade, stage, and histology, but the associations vary widely across the literature [38,39,40,41]. Notably, no prior research has explored the associations between these factors and adverse prognostic factors in endometrial cancer such as lymphovascular space invasion (LVSI), microcystic, elongated and fragmented pattern of myometrial invasion (MELF), or microsatellite instability (MSI). LVSI is the presence of cancer in lymphatic and/or vascular spaces within the uterine myometrium and is an independent risk factor for endometrial cancer recurrence [42]. MELF is associated with adverse histological findings such as larger tumour size, deeper myometrial invasion, and LVSI [43]. MSI serves as an indicator of a faulty DNA mismatch repair system and plays a significant role in the progression of multiple cancer types [44]. MSI-high or positive status was reported to correlate with advanced histological parameters such as deep myometrial invasion and higher grade endometrioid-type adenocarcinomas [44].

There is a growing body of evidence indicating adipocytokines and IGFs are physiologically linked and work synergistically to modulate cancer risk via interconnected pathways, summarised in Figure 1 [45,46,47,48,49]. Adiponectin, TNFα, and IL6 influence each other, with adiponectin reducing TNFα activity, but TNFα and IL6 inhibiting adiponectin production [38,39,40]. Low adiponectin levels promote the production of pro-inflammatory cytokines, fostering a permissive tumour microenvironment, facilitating tumourigenesis. The cytokines also stimulate each other; TNFα induces IL6 expression and release [50]. Obesity-associated insulin resistance leads to decreased adiponectin levels, elevated insulin, and bio-available IGF-I levels. Adiponectin inhibits IGF1 signalling in certain cell types, reducing cell proliferation. IGF1 can directly suppress serum adiponectin levels and stimulate leptin production [51]. Additionally, IL6 and TNFα expression are higher in obese individuals with insulin resistance, with TNFα playing a role in insulin resistance [52].

Our main objective was to evaluate the levels of adiponectin, leptin, TNFα, IL6, and IGFs 1 and 2 in endometrial cancer patients compared to control patients with benign gynaecological conditions and to assess their relationship with obesity using the WHO BMI classification [57]. Additionally, we aimed to explore the correlation between these markers and tumour characteristics such as grade, stage, histology, LVSI, MELF, and MSI. We also assessed the effect of treatment (surgery with or without adjuvant treatment) on the markers by comparing baseline and follow-up marker levels on day 1 and at 6 months post-surgery. Given the absence of established biomarkers for endometrial cancer, studying these markers and their changes post-surgery may provide valuable prognostic insights. We followed the REMARK reporting guidelines for presenting all relevant information throughout the study [58].

## 2. Materials and Methods

### 2.1. Ethics Approval and Participant Recruitment

The study obtained approval from the Health Research Authority and Health and Care Research Wales, UK. It included 50 consecutive endometrial cancer patients (study group) aged 34–95 years, and 50 patients with benign gynaecological conditions (control group) aged 36–90 years, referred to the Royal Surrey NHS Foundation Trust, Guildford, UK. Control patients were age-matched with study patients by decades.

Demographic data, including age, parity, BMI, smoking history, HRT and hormonal contraception use, diabetes, hypertension, prior cancer history, tamoxifen use, and family history of endometrial cancer, were collected for both groups. Additional clinical and histological information were obtained from medical records.

Endometrial cancer patients underwent Da Vinci robotic-assisted hysterectomy, bilateral salpingo-oophorectomy, peritoneal washings, and sentinel node dissection, or dissection of pelvic and para-aortic lymph nodes. Twenty-six patients had surgical follow up only, whereas eleven patients received radiotherapy only, and thirteen patients received chemotherapy and radiotherapy post-surgery. None of the patients demonstrated any sign of disease progression at the 6-month follow-up.

### 2.2. Plasma Preparation and Storage

Blood samples from the study group were collected at three time points: before surgery (baseline, day 0), one day after surgery (day 1), and six months after surgery. The patients were instructed to fast overnight for blood collection. The control group had blood collected once during their clinic appointment. EDTA-treated Vacutainer tubes (30 mL) were used for blood collection. Plasma was separated by centrifuging the samples immediately after collection at 2400 g for 10 min. The separated plasma was then pipetted into cryotubes and stored at −80 °C until processed. Plasma from the same patient was stored in multiple 1.5 mL cryotubes to avoid repeated freeze–thaw cycles.

### 2.3. Enzyme Linked Immunosorbent Assay (ELISA)

Enzyme linked immunosorbent assay (ELISA) was performed at room temperature, as per the manufacturer’s instructions; Adiponectin (#DY1065), Leptin (#DY398), IGF1 (#DY291), IGF2 (#DY292), IL6 (#DY206), TNFα (#DY210)—all from R&D Systems, NE Minneapolis, MN, USA. Samples were diluted 1000-fold for adiponectin estimation and 100-fold for leptin estimation using the reagent diluent provided in the ELISA kits. For IGF1 and 2, acid-ethanol treatment at room temperature was used to release the analytes from IGF binding proteins (IGFBPs), as they are frequently bound to IGFBP in the plasma, which may result in a lower estimated value. Reagents for plasma sample pretreatment comprised an acid–ethanol extraction solution, involving the addition of 2.5 mL of 37% hydrochloric acid to 10 mL of deionised or distilled water, followed by ethanol to reach a final volume of 100 mL. 30 μL of plasma was added to 120 μL of the acid–ethanol extraction solution, incubated for 30 min, and then centrifuged for 5 min at 10,000 rpm. Thereafter, 100 μL of the supernatant was transferred to another polypropylene tube containing 200 μL of 2M Tris buffer (pH of 7.6). Following this, 300 μL of reagent diluent was added and mixed, and the ELISA was conducted promptly. Due to sample pretreatment for the release of the IGFs, the concentration obtained from the standard curve required adjustment by a dilution factor of 30.

For ELISA, both study and control samples were run on the same high-binding microplates (Greiner Bio-one, #675061, Kremsmünster, Austria) and run in triplicate. The plates were incubated overnight with the respective capture antibodies. The rest of the steps were carried out the next day with three washes with Phosphate Buffer Saline (PBS) solution between each step. The plates were successively incubated with reagent diluent for one hour, sample/standard for two hours, detection antibody for two hours, followed by incubation with Streptavidin-Horseradish Peroxidase conjugate for 20 min, and finally the chromogenic substrate 3,3′, 5,5′-tetramentylbenzidine (Thermo Scientific™ 1-Step™ TMB ELISA Substrate Solutions, #34029, Waltham, MA, USA) for 20 min for colour development. Thereafter, without any further wash, stop solution and 2N sulphuric acid (R&D systems, #DY994) were added to the chromogen containing wells to stop further reaction, and the absorbance of the solution in the wells was measured immediately at a 450 nm wavelength, with a correction wavelength set at 540 nm, using a spectrophotometer. Concentrations of biomarkers in the plasma were determined and corrected using the appropriate dilution factor by comparing the absorbance values with the standard curve generated for each microplate. All ELISAs were performed by the same person in a blinded fashion.

### 2.4. Assessment of Body Fat Distribution

**Figure 3:**
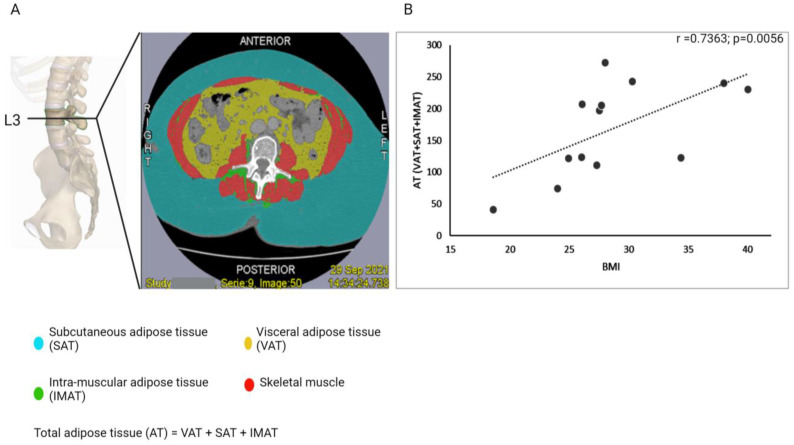
(**A**) The image shows a contrast CT scan at the L3 vertebra level, depicting adipose tissue (AT) distribution. AT is measured as a combination of subcutaneous adipose tissue (cyan, SAT), visceral adipose tissue (yellow, VAT), and intramuscular adipose tissue (green, IMAT). Skeletal muscle is shown in red. (**B**) The scatter plot shows the correlation between BMI and total adipose tissue (AT) distribution, as measured at the L3 vertebra level, calculated using Spearman’s correlation test for 13 patients. r represents the Spearman correlation coefficient, and p represents the associated *p*-value, with *p* < 0.05 significant.

We assessed total adipose tissue (AT) distribution in 13 endometrial cancer patients with contrast CT scans using Tomovision SliceOmatic 5.0 software. AT included visceral adipose tissue (VAT), subcutaneous adipose tissue (SAT), and intramuscular adipose tissue (IMAT) at the L3 vertebral level (Figure 3). To evaluate comparability, we correlated these AT measurements with BMI in the same 13 patients using Spearman’s correlations.

### 2.5. Statistical Analysis

Statistical analysis was conducted using Microsoft Excel, GraphPad Prism 9, G*power 3.1.9.7, and R Studio (version 2023.09.0+463) software.

As no study has assessed all six markers together in a cohort of endometrial cancer patients in the Surrey region of the UK prior to this study, we consider this study a pilot study. We performed a post hoc power calculation using ‘G*power software’. The post hoc calculation revealed that with a moderate effect size (f^2^ = 0.15) and a total sample size of 100, the multivariate linear regression comparing our study (*n* = 50) and the control population (*n* = 50) has high statistical power (94%).

Descriptive statistics summarised demographic, clinical, and histological characteristics for both the study and control populations. Fischer’s exact test assessed compatibility or differences in demographic and clinical features between the two groups. 

To compare biomarker levels between study and control populations, a linear regression model was used, adjusting for BMI, diabetes, and parity, which are the unmatched factors between the study and control groups. This adjustment aimed to mitigate the confounding impact of these factors, considering their individual influence on the risk of endometrial cancer. The inclusion of adjustment for BMI in the regression model is particularly crucial due to its established strong correlations with adiponectin and leptin levels, potentially introducing confounding effects into the results. The effects of demographic characteristics on biomarker levels were evaluated with populations divided into binary groups for ease of comparison. Univariate linear regression was initially performed, followed by multivariate regression using the significant associations from the univariate analysis. For cancer patients, the effects of tumour grade, stage, histology, LVSI, MELF, and MSI on the biomarkers were assessed using multivariate linear regression, with binary categorisations to facilitate comparisons. All analyses were performed using the functions ‘lm’ and ‘Anova’ from ‘stats’ and ‘car’ R packages, respectively.

Longitudinal data analysis employed mixed model ANOVA, considering time point, BMI, and their interaction (degrees of freedom = 2). Analyses were performed using the functions ‘lmer’ and ‘anova’ from the ‘lmerTest’ package.

Plasma biomarker levels were categorised into quartiles, and a logistic regression model was used to calculate odds ratios (ORs) and 95% confidence intervals (95% CIs) for risk assessment, using the lowest quartile as the reference group. We adjusted the calculations for BMI, diabetes, and parity. Analyses were performed using the ‘glm’ function from ‘stats’ R package.

A significance level of *p* < 0.05 was considered statistically significant throughout the study. Adjustments to the *p*-values for multiple comparison were performed using the Benjamini and Hochberg approach in ‘R’ and are represented in the text as adjusted *p*-values.

## 3. Results

### 3.1. Demographic Characteristics

The demographic characteristics of both groups are shown in Table 1. The mean age of the study group at the time of diagnosis was 65.7 years (range 34–95 years), and that of the control group was 66.1 years (range 36–90 years). The two populations were well-matched for all criteria except BMI distribution, the number of nulliparous women, prevalence of diabetes, and usage of HRT.

### 3.2. BMI as an Indicator for Obesity

BMI is considered a flawed measure of obesity due to its limited consideration of weight distribution and muscle-to-fat ratio. Consequently, measuring sarcopenic obesity is preferred over relying solely on BMI because it offers a more comprehensive evaluation of body composition, encompassing both muscle and fat mass [59]. As previously mentioned, we assessed total adipose tissue (AT) distribution at the L3 vertebral level in 13 patients with CT scans and calculated the correlation between BMI and AT measurements to determine if BMI could serve as a surrogate indicator for body adipose tissue distribution (Figure 3A). Out of the 13 patients in this group, 10 exhibited greater distribution of visceral fat (VAT) compared to intramuscular fat (IMAT). This pattern suggests the presence of visceral adiposity, a type of fat distribution often linked to insulin resistance and heightened metabolic risk [60]. Although our sample size is limited, this observation hints at a higher prevalence of metabolically obese patients who are commonly associated with endometrial cancer. Spearman correlation analysis revealed a strong correlation between BMI and AT measurements (r = 0.7363, *p* = 0.006) (Figure 3B), affirming the use of BMI as an indicator of obesity in our study.

### 3.3. Endometrial Cancer Tissue Characterisation in Study Patients

Endometrial cancer is characterised by its grade, stage, and histology among the study population (shown in Table 2). In our study, most of the patients presented with/had grade 1 endometrial cancer (46%) rather than grades 2 (28%) or 3 (26%). Also, more patients were in Stage I (82%), compared to Stage II (4%) and Stage III (14%) (staging of endometrial cancer as per the International Federation of Gynecology and Obstetrics (FIGO) 2009 classification [61],(Supplementary Materials, Table S5). As for histology, 72% had type 1 cancer compared to 28% with type 2 (Bokhman classification [62]). Type 1 endometrial cancers were all of the endometrioid variety. The type 2 endometrial cancers included the following subtypes—serous, mucinous, and carcinosarcoma. Grade 3 endometrioid endometrial cancer was included in type 2 histology, as it demonstrated more clinical and immunohistochemical alignment with type 2 endometrial cancer rather than type 1 [63]. LVSI was present in 30% of the cancer patients, MELF in 17% of grade 1/2 cancers, and MSI was detected in 24% of the reported cases.

This distribution is consistent with endometrial cancer statistics in other studies [43,64,65].

### 3.4. Comparison of the Levels of Adipocytokines and IGF in Study and Control Populations

**Figure 4:**
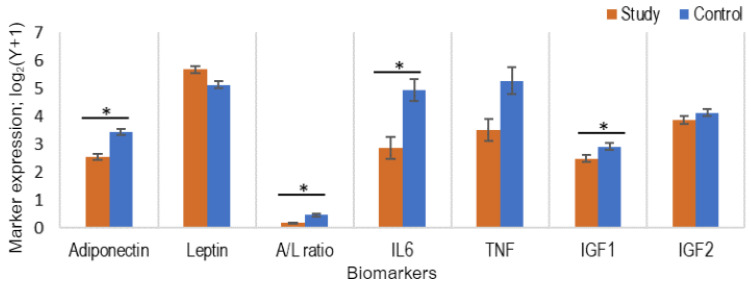
Bar diagrams comparing the levels of all biomarkers between the study population (baseline values at time-point 1, *n* = 50) and control population (*n* = 50) using linear regression, adjusted for BMI, diabetes, and parity. * denotes a significant difference (*p* < 0.05).

We conducted a comparative analysis of baseline marker levels between the study and control groups, including adiponectin, leptin, IL6, TNFα, IGF1, and IGF2, while also adjusting for BMI, parity, and diabetes (Figure 4). A significantly lower level of adiponectin was found in the study population throughout, with a mean of 5.9 µg/mL (range 0.8–27.2 µg/mL), than in the control population with a mean of 11.0 µg/mL (range 3.1–29.6 µg/mL) (*p* < 0.0001; adjusted *p* < 0.0001). Conversely, plasma leptin levels were significantly higher in the study group, averaging 61.2 ng/mL (range: 14.2–222.7 ng/mL), compared to the control group with an average of 41.9 ng/mL (range: 8.9–135.6 ng/mL), but this difference was not significant after adjusting for BMI, diabetes, and parity (*p* = 0.240; adjusted *p* = 0.723). We have also calculated the A/L ratio, indicative of adipose tissue function, and found it significantly lower in the study group (mean ratio: 0.14) than in the control group (mean ratio: 0.43) (*p* < 0.0001; adjusted *p* = 0.0005), signifying adipose tissue dysfunction [20].

Among the cytokines, plasma IL6 was found to be significantly lower in the study population, averaging 17.3 pg/mL (range 2.2–51.2 pg/mL), compared to the control population with an average of 19.3 pg/mL (range 4.2–54.7 pg/mL) (*p* = 0.0003; adjusted-*p* = 0.002). TNFα levels ranged from 0–3504 pg/mL (mean 118.8 pg/mL) in the study population and 0–5182 pg/mL (mean 418.8 pg/mL) in the control population, and hence similar to IL6, TNFα was also lower in the study population than the control population; however, the difference was not significant when the *p*-value was adjusted (*p* = 0.021; adjusted-*p* = 0.083).

As for the growth factors, plasma IGF1 levels were lower in the study population, with a mean value of 5.8 ng/mL (range = 0.0008–19.8 ng/mL), than in the control population with a mean value of 7.8 ng/mL (range = 0.9–26.0 ng/mL). However, the difference was not significant (*p* = 0.350; adjusted *p* = 0.723). Also, no significant difference was noted between IGF2 levels in the study (range 2.2–51.2 ng/mL, mean 17.3 ng/mL) and control populations (range 4.2–54.7 ng/mL, mean 19.3 ng/mL) (*p* = 0.270; adjusted *p* = 0.723).

Therefore, cancer patients had significantly lower levels of adiponectin, IL6, and a lower A/L ratio compared to control patients after adjustment for BMI, diabetes, and parity. Comparisons of biomarkers levels and sub-groups related to BMI, diabetes, menopause and parity have been included in the Supplementary Materials (Figures S1–S4, respectively). 

### 3.5. Comparison of Biomarker Levels at Different Time Points in the Study Population (Pre-Surgery, Day 1 Post-Surgery, and 6 Months Post-Surgery)

**Figure 5:**
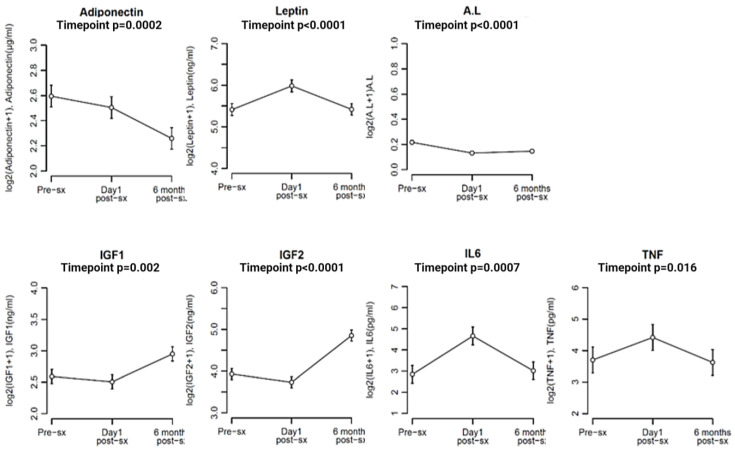
Line diagrams demonstrating the levels of all biomarkers in the study population at three different timepoints—pre-surgery, one day post-surgery, and 6 months post-surgery (adjusted for BMI). X axis—log of levels of biomarkers, Y axis—three time-points. Error bars represent standard error of mean. Sx—surgery. Calculations performed using mixed model ANOVA in ‘R’, considering time point, BMI, and their interaction (degrees of freedom = 2) with a sample size of 50 in each group of time-point.

The levels of adiponectin in the study population decreased progressively across the three time points—pre-surgery, one day post-surgery, and 6 months post-surgery, with a statistically significant reduction observed between pre-surgery levels and 6 months post-surgery (*p* = 0.0002; adjusted *p* = 0.0007). On the other hand, leptin levels were significantly higher (*p* < 0.0001; adjusted *p* < 0.0001) one day post-surgery than at pre-surgery or 6 months post-surgery, with no significant difference being observed between pre-surgery and 6-month post-surgery levels. The A/L ratio significantly decreased (*p* < 0.0001; adjusted *p* < 0.0001) from pre-surgery to one-day post-surgery levels, reflecting the rise in leptin levels, with no significant difference between one day post-surgery and 6 months post-surgery (Figure 5).

IL6 levels were significantly higher (*p* = 0.0007; adjusted *p* = 0.002) one day post-surgery compared to pre-surgery levels in the study population and decreased to pre-op levels at 6 months post-surgery. TNFα levels were significantly high (*p* = 0.016; adjusted *p* = 0.016) at one day post-surgery, but no significant difference was found between pre-surgery levels and 6-month post-surgery levels (Figure 5).

IGF1 and IGF2 levels significantly increased (*p* = 0.002 and *p* < 0.0001 and adjusted *p* = 0.004 and <0.0001, respectively) at 6 months post-surgery compared to pre-surgery and one day post-surgery levels (Figure 5).

Given the significant changes in adiponectin and IGF levels at 6 months post-surgery, we analysed the variations in these three markers between patients with surgical follow-up only and those who underwent adjuvant radiotherapy or chemo-radiotherapy. Patients without adjuvant treatment exhibited a significant decrease in adiponectin levels at 6 months post-surgery (*p* < 0.0001; adjusted *p* = 0.0002). However, in patients receiving adjuvant treatment (radiotherapy or chemo-radiotherapy), although a decreasing trend was observed at 6 months, the decline was not statistically significant post-radiotherapy (*p* = 0.414; adjusted *p* = 0.827) and chemo-radiotherapy (*p* = 0.235; adjusted *p* = 0.235).

Regarding IGFs 1 and 2, patients without any adjuvant treatment showed significant increases in levels at 6 months, although the adjusted *p*-values were not significant (*p* = 0.014 and 0.023, respectively; adjusted *p* = 0.069 and *p* = 0.091, respectively). In contrast, in patients receiving adjuvant radiotherapy or chemo-radiotherapy, a significant rise in IGF2 levels was noted at 6 months post-radiotherapy (*p* = 0.0009; adjusted *p* = 0.007) and post-chemo-radiotherapy (*p* = 0.002; adjusted *p* = 0.017), while the increase in IGF1 levels was not significant in the adjuvant treatment group—radiotherapy (*p* = 0.188; adjusted *p* = 0.754) and chemo-radiotherapy (*p* = 0.017; adjusted *p* = 0.069), though the upward trend persisted.

### 3.6. Association between Levels of Biomarkers and Demographic Characteristics of Both Populations

We analysed the biomarker levels in relation to demographic characteristics of both cancer and control patients, including BMI, age, ethnicity, parity, use of HRT, use of hormonal contraception, diabetes, and hypertension. Initially, a univariate analysis was conducted, followed by *p*-value adjustment (Supplementary Materials, Tables S1 and S2, Figure S5). Thereafter, a multivariate analysis was performed using the associations found to be significant at the univariate analysis (Supplementary Materials, Figure S6).

Adiponectin levels exhibited an inverse correlation with BMI, with lower levels observed in overweight or obese patients than in normal-weight patients in both populations, although it was significant only in the control population (*p* = 0.004). Conversely, leptin levels were positively correlated with BMI and were higher in overweight and obese patients compared to normal-weight patients in both study (*p* = 0.09) and control groups (*p* = 0.0002). Consequently, the A/L ratio was lower in overweight or obese patients in both groups (study, *p* = 0.012; control, *p* < 0.0001), following a pattern similar to adiponectin. BMI had no significant associations with IL6, TNFα, IGF1, and IGF2.

Adiponectin levels were higher in patients aged ≥60 years in both study (*p* = 0.026) and control (*p* = 0.496) populations. The A/L ratio followed a similar trend to adiponectin and was higher in patients aged ≥60 years in both study (*p* = 0.009) and control (*p* = 0.871) populations. Leptin, however, was higher in the younger patients (<60 years) for both populations, although this finding was not significant after multivariate analysis. IGF1 and IGF2 were higher in patients aged ≥60 years in the study group (*p* = 0.083 and *p* = 0.673, respectively) but conversely higher in younger patients in the control population (*p* = 0.711 and *p* = 0.069, respectively). No associations were observed between age and IL6 or TNFα.

IL6 levels demonstrated elevated levels in nulliparous women (study group, *p* = 0.047).

Corresponding to the age trend, adiponectin demonstrated higher levels in menopausal women (study group *p* = 0.065, control group *p* = 0.014); the A/L ratio was also elevated in menopausal women (study group *p* = 0.083, control group *p* = 0.005); conversely, leptin was more abundant in pre-menopausal women (study group *p* = 0.208, control group *p* = 0.006). For menopausal women within the study group, IGF1 and IGF2 levels were elevated (*p* = 0.069 and 0.356 respectively), whereas the control group displayed lower levels of both IGFs in menopausal women (*p* = 0.019 and 0.361, respectively). Thus, only in the case of the IGFs, menopause affected the marker levels in an opposite manner between the study and control groups, although the differences were not significant for IGF2.

Moreover, among non-diabetic women, IGF1 levels surpassed those of diabetic women (study group *p* = 0.423, control group *p* = 0.009). Therefore, diabetes appears to play a role in modulating IGF1 levels, although the results were not significant for the study population.

No significant associations were found between hypertension, HRT, hormonal contraception intake, history of breast cancer, tamoxifen intake, family history of Lynch syndrome, and the various adipocytokines and IGFs in either population. Consequently, the results of these associations are not documented here.

### 3.7. Associations between Levels of Biomarkers and Cancer Characteristics in the Study Population

Endometrial cancer exhibits distinct molecular and genetic profiles with some still unclassified molecular profiles. Correlating biomarkers with cancer characteristics allows for a more accurate diagnosis and classification of cancer subtypes. In our study, we correlated the levels of the six markers with various endometrial cancer characteristics with known prognostic significance, such as grade, stage, histology, LVSI, MELF, and MSI. Initially, we conducted a univariate analysis and *p*-value adjustment (Supplementary Materials, Table S3, Figure S5), followed by a multivariate analysis of significant factors (Supplementary Materials, Figure S7).

Adiponectin exhibited an inverse relationship with LVSI, with significantly lower levels in patients without LVSI (*p* = 0.033). On univariate analysis, leptin levels showed significantly higher values in type 1 endometrial cancer compared to type 2 (*p* = 0.035) and in patients without MELF (*p* = 0.022). However, these associations did not reach significance in the multivariate analysis (*p* = 0.258 and *p* = 0.084, respectively). TNFα levels were noted to be higher in patients with MELF (*p* = 0.015). No significant associations were found between grade, stage, or MSI with any of the markers.

### 3.8. Endometrial Cancer Risk Assessment

**Figure 6:**
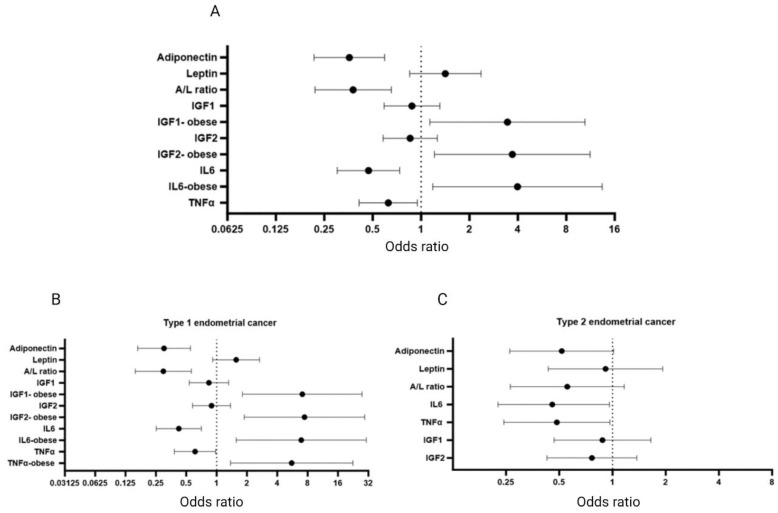
Risk association between all six markers and endometrial cancer calculated using logistic regression: (**A**) all endometrial cancer patients (*n* = 50); (**B**) type 1 endometrial cancer (*n* = 36); (**C**) type 2 endometrial cancer (*n* = 14). The dots indicate the odds ratio (OR) and the bars indicate the 95% confidence interval (CI) plotted after adjusting for BMI, diabetes, and parity. OR < 1: inverse correlation, OR > 1: positive correlation, OR = 1 or 95% CI spans 1: no significant association. A correlation analysis among the six biomarkers themselves using Pearson’s correlation is available in the Supplementary Materials, Figure S8 and an explanation of Pearson’s correlation values is provided in Supplementary Materials, Table S4.

To estimate the risk of endometrial cancer for each biomarker, we calculated the odds ratio, adjusted for BMI, diabetes, and parity (Figure 6A). Adiponectin (OR_Q4 vs. Q1_ = 0.358, 95% CI 0.216–0.594, *p* < 0.0001; adjusted *p* = 0.0002) and the A/L ratio (OR_Q4 vs. Q1_ = 0.378, 95% CI 0.219–0.653, *p* = 0.0001; adjusted *p* = 0.0009) exhibit a significant inverse association with the risk of endometrial cancer. It is noteworthy that we find that leptin is associated with increased risk of endometrial cancer in diabetic patients only (OR_Q4 vs. Q1_ = 3.787, 95% CI 0.903–15.876, *p* = 0.069), suggesting a role for leptin in elevating the risk of endometrial cancer in diabetic individuals. IL6 is positively associated with endometrial cancer risk in the obese population (OR_Q4 vs. Q1_ = 3.979, 95% CI 1.184–13.375, *p* = 0.026), while the same is not true when the entire population is considered (OR_Q4 vs. Q1_ = 0.471, 95% CI 0.301–0.736, *p* = 0.001; adjusted *p* = 0.007). This suggests that IL6′s role in endometrial cancer pathophysiology is likely modulated by obesity. TNFα increased the risk of endometrial cancer in the diabetic population (OR_Q4 vs. Q1_ = 4.176, 95% CI 0.990–17.609, *p* = 0.052), whereas this effect is not seen in the entire population (OR_Q4 vs. Q1_ = 0.625, 95% CI 0.412–0.949, *p* = 0.024; adjusted *p* = 0.096). IGF1 and IGF2 do not show significant associations with the risk of endometrial cancer in the entire population. However, both markers increase the risk of endometrial cancer in obese patients (OR_Q4 vs. Q1_ = 3.455, 95% CI 1.135–10.459, *p* = 0.029, and OR_Q4 vs. Q1_ = 3.694, 95% CI 1.213–11.247, *p* = 0.021, respectively). This brings into focus the complex interplay between obesity and diabetes and markers such as leptin, IL6, TNFα, and IGFs in elevating the risk of endometrial cancer.

Given that type 1 endometrial cancer is primarily linked to obesity, while type 2 endometrial cancer is non-obesity related, we examined the risk profiles of all the markers separately for these two types (Figure 6B and Figure 6C, respectively). In type 1 endometrial cancer, the risk was inversely related to adiponectin (*p* < 0.0001; adjusted *p* = 0.0005), the A/L ratio (*p* = 0.0002; adjusted *p* = 0.001), IL6 (*p* = 0.001; adjusted *p* = 0.007), and TNFα (*p* = 0.040; adjusted *p* = 0.172). Notably, IL6 and TNFα were positively associated with type 1 endometrial cancer in obese patients only (*p* = 0.011 and 0.016, respectively). Leptin and IGF 1 and 2 were not linked to endometrial cancer risk, except for IGF1 and 2 increasing the risk of type 1 endometrial cancer in obese individuals (*p* = 0.005 and 0.004, respectively).

Concerning type 2 endometrial cancer, adiponectin maintained an inverse relation with the risk, although not reaching significance (*p* = 0.056; adjusted *p* = 0.07). IL6 (*p* = 0.039; adjusted *p* = 0.262) and TNFα (*p* = 0.040; adjusted *p* = 0.168) still exhibited an inverse correlation with type 2 endometrial cancer, although there was no difference in the risk profile by weight category. None of the other associations were significant for type 2 endometrial cancer.

## 4. Discussion

Obesity is a well-established risk factor for the development of endometrial cancer. It is believed that chronic inflammation and insulin resistance resulting from obesity contribute to the increased susceptibility to endometrial cancer [66]. While previous research has explored the link between adipocytokines and endometrial cancer, limited attention has been given to the role of IGFs or the combined impact of adipocytokines and IGFs [24,67,68,69,70]. Our research aims to fill these knowledge gaps, offering insights into endometrial cancer development and potential diagnostic and prognostic indicators. 

In our study, we observed that adiponectin levels were significantly lower in the cancer group compared to the control group, even after accounting for BMI. This finding aligns with similar observations made by Erdogan et al. [71] and Petridou et al. [72]. Low adiponectin levels are regarded as a risk factor for endometrial cancer, a view supported by Cust et al. [12], who noted an inverse relationship between adiponectin levels and endometrial cancer risk. In our study, low adiponectin levels posed a 35.8% higher endometrial cancer risk, even after adjusting for BMI, diabetes, and parity. Given this association, there is growing interest in strategies to raise adiponectin levels through interventions such as peroxisome proliferator-activated receptor gamma (PPARγ) agonists or vigorous aerobic exercise as potential therapeutic approaches in obesity-related diseases and cancers [73]. Contrarily, the loss of significance in the inverse risk association between adiponectin and endometrial cancer when considering only type 2 endometrial cancers could be attributed to the general perception that type 2 endometrial cancer is obesity independent. It might also be influenced by the smaller sample size of type 2 cancers within our study group; however, this observation requires validation in a larger cohort of type 2 endometrial cancer.

Furthermore, our study uncovered an intriguing association between adiponectin levels and LVSI in endometrial cancer patients. Individuals with LVSI exhibited higher adiponectin levels compared to those without LVSI, although their levels were still lower than those in the control group. This finding may seem paradoxical, as LVSI is typically considered an adverse prognostic factor. One study by Ueno et al. has suggested that elevated adiponectin levels are linked to the absence of LVSI in endometrial cancer tissues, implying a potential protective role of adiponectin against lymphovascular invasion [74]. However, contrasting findings exist, as demonstrated by Terlikowska et al., who found no statistically significant correlation between adiponectin levels and LVSI [75]. Hence, this apparent inverse relationship between adiponectin and LVSI warrants further investigation to determine its pathophysiological and clinical significance.

Leptin levels were found to be higher in endometrial cancer patients compared to the control group, but this was not significant when adjusted for BMI, parity, and diabetes. Leptin levels were found to be significantly higher in overweight and obese patients compared with normal-weight patients in both populations. This is consistent with leptin levels being strongly influenced by BMI. Cymbaluk et al. reported similar findings, highlighting that average leptin levels are higher in endometrial cancer patients and positively correlated with BMI [76]. Given the profound impact of BMI on leptin levels and the prominent role of obesity in endometrial cancer, leptin may not be an ideal candidate as a diagnostic or prognostic marker for this cancer. However, treatments aimed at reducing elevated leptin levels, such as Metreleptin, leptin-sensitising medications, soluble leptin receptors, and leptin receptor antagonists, may complement efforts to raise adiponectin levels in the treatment of obesity-related diseases [77,78,79].

The adiponectin-to-leptin (A/L) ratio is considered an indicator of adipose tissue dysfunction, with a lower ratio being associated with an increased risk of obesity-related diseases [80,81]. Our study confirmed this association, demonstrating that similar to adiponectin, a low A/L ratio was associated with a 37.8% increased risk of endometrial cancer. Therefore, strategies aimed at improving the A/L ratio by concurrently elevating adiponectin and reducing leptin levels may be a more effective approach for the management of endometrial cancer.

In our investigation of IL6 and TNFα, we found that baseline IL6 levels were significantly lower in the cancer group compared to the control group, even when adjusted for BMI, diabetes, and parity. This finding contrasts with some previous studies; for example, Bellone et al. reported higher IL6 levels in endometrial cancer patients, particularly in those with uterine papillary serous carcinoma, a more aggressive type of type 2 endometrial cancer [65]. Chopra et al., however, found no significant elevation in IL6 levels in endometrial cancer patients compared to healthy controls [25]. Similarly, TNFα baseline levels were higher in the control group in our study after adjusting for BMI, diabetes, and parity, which differs from studies by Friedenreich et al. and Wang et al., who found no significant differences in levels of both markers between the study and control groups [82,83]. Although the relationship between IL6 and TNFα levels and endometrial cancer appears contentious in the literature, both cytokines have been shown to promote endometrial cancer progression by facilitating cell growth, metastasis, and epithelial-mesenchymal transition (EMT) [37]. Interestingly, our study noted that both IL6 and TNFα levels were significantly higher in the control population compared to the cancer population, although the significance was lost when the adjusted *p*-value was used for TNFα. This counterintuitive finding may be influenced by various factors, such as a smaller sample size and the heterogenous stages of the study population with a prevalence of initial stages of disease. Additionally, other factors affecting cytokine levels in the plasma, such as stress, inflammation, diurnal variation, and metabolic diseases, may also influence the levels of these markers [84]. The association between IL6 and stress-related hormones, such as adrenocorticotropic hormone (ACTH) and cortisol, could potentially explain the differences in IL6 levels between cancer and control patients [84]. Additionally, a positive association between cytokines in the obese patients with type 1 endometrial cancer was noted, which was not observed in type 2 endometrial cancers. This underscores the distinction between the two types of endometrial cancer and emphasises the significance of obesity-related inflammation in the pathogenesis of type 1 endometrial cancer.

It is important to emphasise that our control group, while cancer-free, cannot be considered entirely healthy because all individuals in the control group have been referred to the hospital due to benign gynaecological conditions, which may have an impact on their inflammatory marker levels. Furthermore, within our study population, there is a smaller representation of higher grade, advanced stage, and type 2 histology, as compared to the predominance of early-grade cancer with a favourable prognosis. This particular distribution may have contributed to the lower levels of IL6 and TNFα in the plasma of the cancer group.

Moving on to the discussion of IGFs, we did not observe significant differences in the levels of both IGF1 and 2 between the cancer and control groups, after adjusting for BMI, diabetes, and parity. It is important to note that IGF1 levels tend to decrease in post-menopausal women due to declining oestrogen levels, a trend that we also observed [85,86]. However, our study highlighted an interesting finding: IGF levels increased after menopause in endometrial cancer patients, suggesting potential modulatory effects of endometrial cancer-related factors on IGF levels. Given the intricate connections between insulin, oestrogen, and the IGF system in the context of endometrial cancer, further exploration of hormonal levels could yield valuable insights. While Petridou et al. reported that endometrial cancer was positively associated with serum levels of IGF2 and inversely associated with IGF1 [36], our study did not establish a significant correlation between IGF levels and endometrial cancer risk. However, intriguingly, our subgroup analysis reveals a positive association between both IGF1 and 2 and an increased risk of endometrial cancer within the obese BMI category, translating to a substantial 3.5 and 3.7-fold increase in cancer risk, respectively. This observation was similarly noted in type 1 endometrial cancer but not in type 2 endometrial cancer. This highlights the importance of obesity in type 1 endometrial cancer and suggests the possibility that, within an obese context, IGFs may contribute to an increased risk of type 1 endometrial cancer. Nevertheless, it is important to acknowledge that the connection between BMI and IGF levels remains controversial in the scientific literature, and hence, this finding warrants further exploration on a larger scale [87].

Our study revealed significant changes in biomarker levels over time following surgery considering the whole cohort of endometrial cancer patients. Adiponectin gradually decreased, while leptin, IL6, and TNFα increased on the first day post-surgery before returning to pre-surgery levels at 6 months. Both IGF1 and IGF2 did not significantly differ between pre-surgery and the first day post-surgery but showed significant increases at the 6-month follow-up compared to baseline. While our study is the first to investigate the impact of endometrial cancer treatment on adipocytokines and IGFs, previous research on different cancer types has shown varying fluctuations in response to surgical stress. For instance, Florescu et al. noted decreased adiponectin levels 24 h after rectal carcinoma surgery, returning to baseline within 7 days [88]. They suggested that surgical stress triggers an adaptive inflammatory process affecting adiponectin levels [88]. Additionally, Shi et al. indicated a potential role of adipose tissue in mitigating the stress response during surgery through adiponectin release [89]. Changes in adiponectin levels have also been linked to factors like radiotherapy, chemotherapy, and immune modulation in cancer patients [90]. In our study, patients without adjuvant treatment exhibited a significant decrease in adiponectin levels at 6 months post-surgery. However, in those receiving adjuvant treatment, although a declining trend was observed at 6 months, it did not reach statistical significance. This discrepancy may be attributed to the unknown mechanisms by which radiotherapy and chemotherapy affect adiponectin levels or the relatively smaller sample size in the adjuvant treatment cohorts, which may lack robustness to assess the impact of different treatments.

Leptin levels exhibit circadian fluctuations and an inverse correlation with cortisol and ACTH, suggesting a role in modulating the body’s response to stress [84]. The increase in post-surgery leptin levels may be attributed to surgical stress, given consistent fasting conditions during sample collection before and on the first day after surgery. Similarly, IL6 and TNFα levels decreased on day one after surgery, likely due to the stress and inflammation associated with surgical procedures.

In our sequential IGF measurements, immediate post-surgery differences were not significant, but we observed notable increases at the 6-month follow-up compared to baseline. This rise was statistically significant in patients without any adjuvant treatment. However, among those receiving adjuvant treatment, the 6-month increase was significant for IGF2 but not for IGF1, although the upward trend persisted for IGF1 as well. Once again, the smaller sample size in the adjuvant treatment cohorts may limit the study’s ability to effectively assess the effects of various treatments.

Holdaway et al. reported a decrease in serum IGF1 and IGF2 levels 24 h after breast cancer surgery, with levels returning to normal within 7 days [91]. While IGF1 reduction was attributed to non-specific surgical effects, the IGF2 decrease remained significant, especially for malignant tumours and larger sizes. Various factors, including growth hormone and insulin-like growth factor binding proteins (IGFBPs), can influence IGF levels, emphasising the need for future research to explore longitudinal IGF assessments alongside other factors like growth hormone, IGFBP, and oestrogen, for a comprehensive understanding of this complex relationship [27].

BMI was re-checked at 6 months post-surgery, and any effect on BMI on 6-month follow-up levels of the markers was accounted for, using mixed model ANOVA calculations. No signs of disease progression were observed in any of the patients at the 6-month post-surgery evaluation. This lack of progression could be attributed to the relatively short follow-up duration, coupled with the majority of patients being in the earlier stages of cancer, thus having a more favourable prognosis. Despite the absence of disease progression, it is premature to definitively assert a disease response. Consequently, longitudinal studies with extended follow-up intervals are essential to comprehensively evaluate the impact of disease response, relapse, and progression on these markers.

In summary, our study provides valuable insights into the complex relationships between adipocytokines, IGFs, and cytokines in endometrial cancer. While we have made significant strides in understanding the associations between these markers and endometrial cancer risk and treatment outcomes, there are still many unanswered questions and areas requiring further investigation. For instance, the paradoxical association between adiponectin and LVSI, the association between menopause and higher IGF levels in endometrial cancer patients, and the potential interplay between these markers in promoting endometrial cancer growth and survival warrant continued research. Despite its limitations, our prospective study design and meticulous data collection provide a robust foundation for future investigations in this critical area of cancer research.

## 5. Conclusions

Our findings indicate that adiponectin levels are influenced by the carcinogenic process independently of BMI, thereby suggesting its potential as a promising marker for endometrial cancer. However, further research with larger prospective studies is necessary to establish the optimal adiponectin threshold associated with protection against endometrial cancer. In addition, this study has unveiled intriguing associations between various biomarkers and cancer characteristics, such as LVSI and MELF. Nevertheless, to comprehensively understand the underlying pathophysiological mechanisms driving these relationships and their significance in the context of new endometrial cancer molecular classification and staging (Supplementary Materials, Table S6), larger-scale investigations are warranted.

Notably, we observed a distinct increasing pattern in the levels of IGFs 1 and 2 following menopause in the study population, in stark contrast to the control group, where these levels typically decreased. This observation raises intriguing questions about the potential influence of endometrial cancer on modulating the IGF system, particularly among menopausal women.

Furthermore, our study revealed a notable post-treatment shift in marker levels at the 6-month mark, with a decrease in all markers except IGFs, which were adjusted for BMI. This distinctive alteration in post-treatment marker patterns, coupled with the higher levels of IGFs observed post-menopause in the cancer patients, suggests potential clinical significance. These findings underscore the imperative need to explore additional factors influenced by endometrial cancer that may play a role in shaping the behaviour of adiponectin and the IGF system.

## Figures and Tables

**Table 1:** Demographic characteristics of the study and control populations. The study and control populations were compared using Fischer’s exact test, with 50 patients in each group, and *p* < 0.05 (*) indicating a significant difference between the two groups. Parity: the number of times a woman has given birth at >24 weeks of gestation; Nulliparous—no live deliveries; BMI: Body Mass Index, BMI = (weight in kg)/(height in m^2^); HRT: Hormone Replacement Therapy.

Parameters			Study Population (*n* = 50)		Control Population (*n* = 50)		*p*-Value
			Number	Percentage	Number	Percentage	
		30–39	3	6%	3	6%	1
Age range (years)		40–49	3	6%	3	6%	
		50–59	11	22%	11	22%	
Mean age (years)		60–69	10	20%	10	20%	
Study	Control	70–79	16	32%	16	32%	
65.7	66.1	80–89	6	12%	6	12%	
		90–99	1	2%	1	2%	
BMI		18.5–24.9	9	18%	20	40%	0.022 *
Average BMI		25.0–29.9	15	30%	19	38%	
Study	Control	≥30.0	26	52%	11	22%	
32.1	26.6						
Parity		P0 (nulliparous)	11	22%	3	6%	0.041 *
		≥P0	39	78%	47	94%	
Ethnicity		Caucasian	43	86%	48	96%	0.112
		Asian	6	12%	1	2%	
		Afro-Caribbean	1	2%	1	2%	
Smoking status		Yes	3	6%	1	2%	0.47
		No	35	70%	33	66%	
		Ex-smoker	12	24%	16	32%	
Menopausal status		Yes	39	78%	42	84%	0.611
		No	11	22%	8	16%	
Hormonal Contraception		Yes	30	60%	32	64%	0.837
		No	20	40%	18	36%	
HRT (menopausal women only)		Yes	10 (*n* = 39)	25.60%	20 (*n* = 42)	47.60%	0.05
		No	29	74.40%	22	52.40%	
Diabetes		Yes	13	26%	3	6%	0.012 *
		No	37	74%	47	94%	
Hypertension		Yes	22	44%	20	40%	0.84
		No	28	56%	30	60%	
Past history of		Yes	6	12%	14	28%	0.078
cancer		No	44	88%	36	72%	
Family history of		Yes	22	44%	28	56%	0.317
cancer		No	28	56%	22	44%	

**Table 2:** Distribution of endometrial cancer characteristics among the study patients (*n* = 50). Endometrial cancer distribution, characterised by grade, stage, histology, LVSI, MELF, MSI. LVSI: Lympho-vascular space invasion; MELF: Microcystic, elongated, and fragmented pattern of invasion, MSI: Microsatellite instability.

Parameter	Sub-Groups		*n* (%)	Parameter	Sub-Groups	*n* (%)
Grade	1		23 (46)	LVSI	Yes	15 (30)
	2		13 (26)		No	35 (70)
	3		14 (28)			
Stage	IA		32 (64)	MELF	Yes	6 (17)
	IB		9 (18)		No	29 (83)
	II		2 (4)			
	III		7 (14)			
Histology	Type 1	Endometrioid	36 (72)	MSI	Yes	10 (24)
	Type 2		14 (28)		No	32 (76)
		Serous	6 (12)			
		Mucinous	1 (2)			
		Endometrioid (G3)	5 (10)			
		Carcinosarcoma	2 (4)			

## Data Availability

The raw data for analyses are not publicly available to preserve individuals’ privacy under the European General Data Protection Regulation. All results generated and methods of analyses are included in this published article and the Supplementary Information File (Supplementary Ray et al.).

## Supplementary Materials

The following supporting information can be downloaded at: https://www.mdpi.com/article/10.3390/cancers16030531/s1. Supplementary Ray et al. for further detailed explanations and analyses. Figure S1: Box and whisker plots illustrating the difference in baseline levels of markers between cancer and control populations (subgroup analysis by BMI). *p* < 0.05 is significant. N = normal weight, OW = overweight, O = obese; Figure S2: Box and whisker plots illustrating the difference in baseline levels of markers between cancer and control populations by using (subgroup analysis by diabetes). *p* < 0.05 is significant. Y = yes, N = no; Figure S3: Box and whisker plots illustrating the difference in baseline levels of markers between cancer and control populations (subgroup analysis by menopause). *p* < 0.05 is significant. Y = yes, N = no; Figure S4: Box and whisker plots illustrating the difference in baseline levels of markers between cancer and control populations by (subgroup analysis by parity). *p* < 0.05 is significant. Y = yes, N = no; Table S1: Levels of the markers with respect to demographic characteristics in the study population; Table S2: Levels of the markers with respect to demographic characteristics in the control population; Table S3: Levels of the markers with respect to endometrial cancer characteristics; Figure S5: Box and whisker plots demonstrating the association (univariate linear regression) between prognostic factors and the biomarkers in study and cancer patients; Figure S6: Box and whisker plots demonstrating the associations between patient demographic factors and the biomarkers in study and control patients using multivariate linear regression (total *n* = 50, sample size for each subgroup have been mentioned in Table 1). X axis- demographic factors’ groups; Y axis- log2 [(level of the marker) +1]. The 25th and 75th percentiles are represented by the lower and upper boundaries of the rectangles, respectively, while the median is indicated by the horizontal line inside the rectangles. The whiskers extend from the box denoting the minimum and maximum values; Figure S7: Box and whisker plots demonstrating the associations between endometrial cancer characteristics and the biomarkers in study population using multivariate linear regression (total *n* = 50, sample size for each subgroup have been mentioned in table 1). X axis- endometrial cancer characteristics’ groups; Y axis- log2 [(level of the marker) +1]. The 25th and 75th percentiles are represented by the lower and upper boundaries of the rectangles, respectively, while the median is indicated by the horizontal line inside the rectangles. The whiskers extend from the box denoting the minimum and maximum values; Figure S8: Association between all 6 markers. (A) Cancer population—at baseline (B) Control population; Table S4: Demonstrating the interpretation of Pearson’s r value; Table S5. FIGO 2009 staging of endometrial cancer (used in the study); Table S6. FIGO 2023 staging of endometrial cancer. References [92,93,94,95,96,97,98,99,100] are cited in the Supplementary Materials.
