# Supplementary material for: Circulating Adipocytokines and Insulin Like-Growth Factors and Their Modulation in Obesity-Associated Endometrial Cancer
Source: Cancers (Basel). 2024 Jan 26;16(3):531. doi: 10.3390/cancers16030531 (PMC10854745; doi:10.3390/cancers16030531)
Supplement: Supplementary file 1 [file cancers-16-00531-s001.zip › cancers-2776432-supplementary.pdf]

Supplementary Material

Ray et al., Circulating adipocytokines and insulin like-growth factors and their modulation in obesity-associated endometrial cancer.

Additional analyses

Comparing the levels of adipocytokines and IGF levels in study and control populations – sub-group analysis

In the course of our subgroup analyses, we uncovered intriguing patterns within our dataset.

We categorised our cancer and control groups by their different BMI categories and used unpaired t-test to compare the levels of the markers between cancer and control groups in each BMI category. We observed that adiponectin remained significantly lower in the control group compared to the cancer group across all BMI categories. In contrast, while leptin levels were initially higher in the cancer group than the control group as mentioned in the main text, this difference was not statistically significant after stratification by BMI (Supplementary Fig. S1).

Furthermore, when we delved deeper into our analysis by examining different subgroups related to diabetes, menopause, and parity, we discovered some noteworthy trends (Supplementary Figs. S2, S3,S4). We used unpaired t-test to compare cancer and control groups in the various categories. Adiponectin levels were significantly lower in cancer patients compared to controls among individuals without diabetes ($p<0.0001$), both pre-menopausal ($p<0.0001$) and post-menopausal patients ($p=0.028$), and multiparous patients ($p<0.0001$). Conversely, leptin levels remained significantly higher in the cancer population compared to controls among those without diabetes ($p=0.002$), and in the menopausal ($p=0.004$) and multiparous patients ($p=0.026$).

Similarly, A/L ratio, IL6 and TNFα levels were significantly lower in the cancer group compared to the control group among the non-diabetic, menopausal, and multiparous women ($p<0.05$). We also noted that IGF 1 was significantly lower in the cancer group compared to the control group in the non-diabetic patients ($p=0.039$), whereas diabetes did not influence IGF2 level. Both IGF1 and 2 were lower in the cancer group compared to control group in the pre-menopausal patients ($p<0.0001$ and $p=0.0006$, respectively), with no notable impact of parity on either marker.

Thus, our findings suggest that factors such as parity and diabetes play a role in modulating the levels of adiponectin in circulation in cancer patients. In contrast, the levels of leptin, IL6 and TNFα and the A/L ratio are influenced by parity, diabetes and menopause. Furthermore, menopause affects both IGF 1 and 2 levels, while diabetes specifically impacts IGF1 levels. For a comprehensive visualization of these observations, please refer to the detailed plots below (Supplementary Figs. S2, S3,S4).

Difference in levels of markers between cancer and control populations (subgroup analysis by BMI)

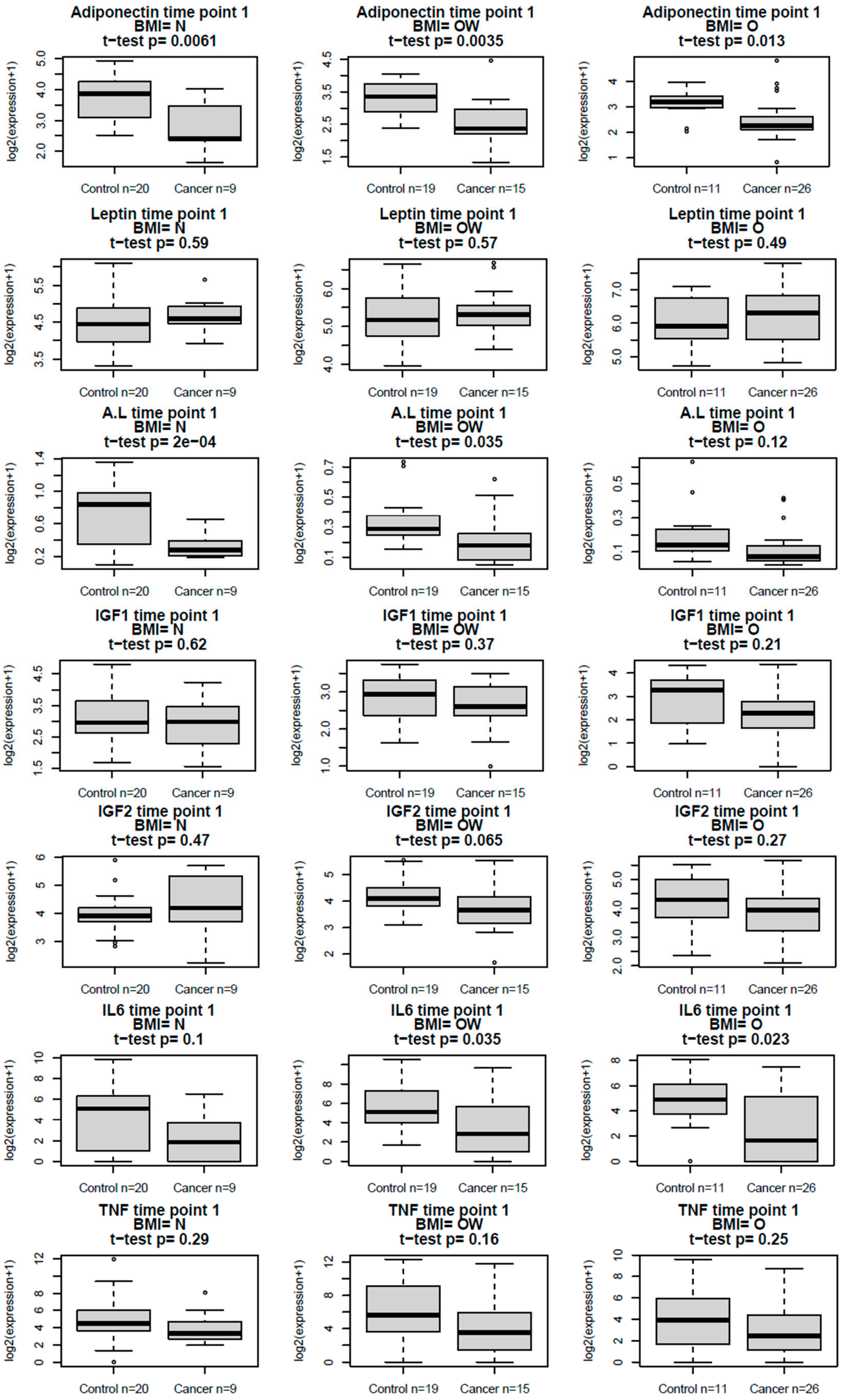

Supplementary Figure S1: Box and whisker plots illustrating the difference in baseline levels of markers between cancer and control populations (subgroup analysis by BMI). p<0.05 is significant. N=normal weight, OW=overweight, O=obese.

Difference in levels of markers between cancer and control populations (subgroup analysis by diabetes)

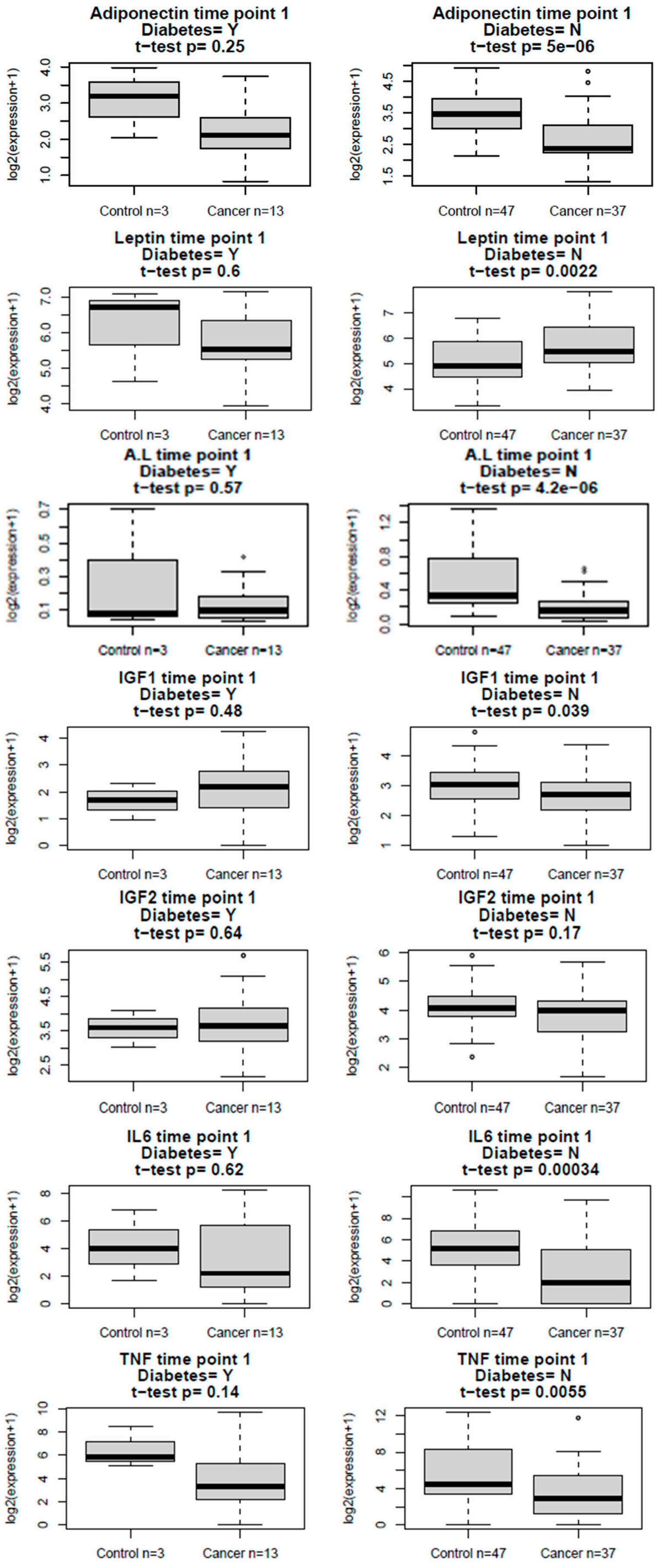

Supplementary Figure S2: Box and whisker plots illustrating the difference in baseline levels of markers between cancer and control populations by using (subgroup analysis by diabetes). $p<0.05$ is significant. Y=yes, N=no.

Difference in levels of markers between cancer and control populations (subgroup analysis by menopause)

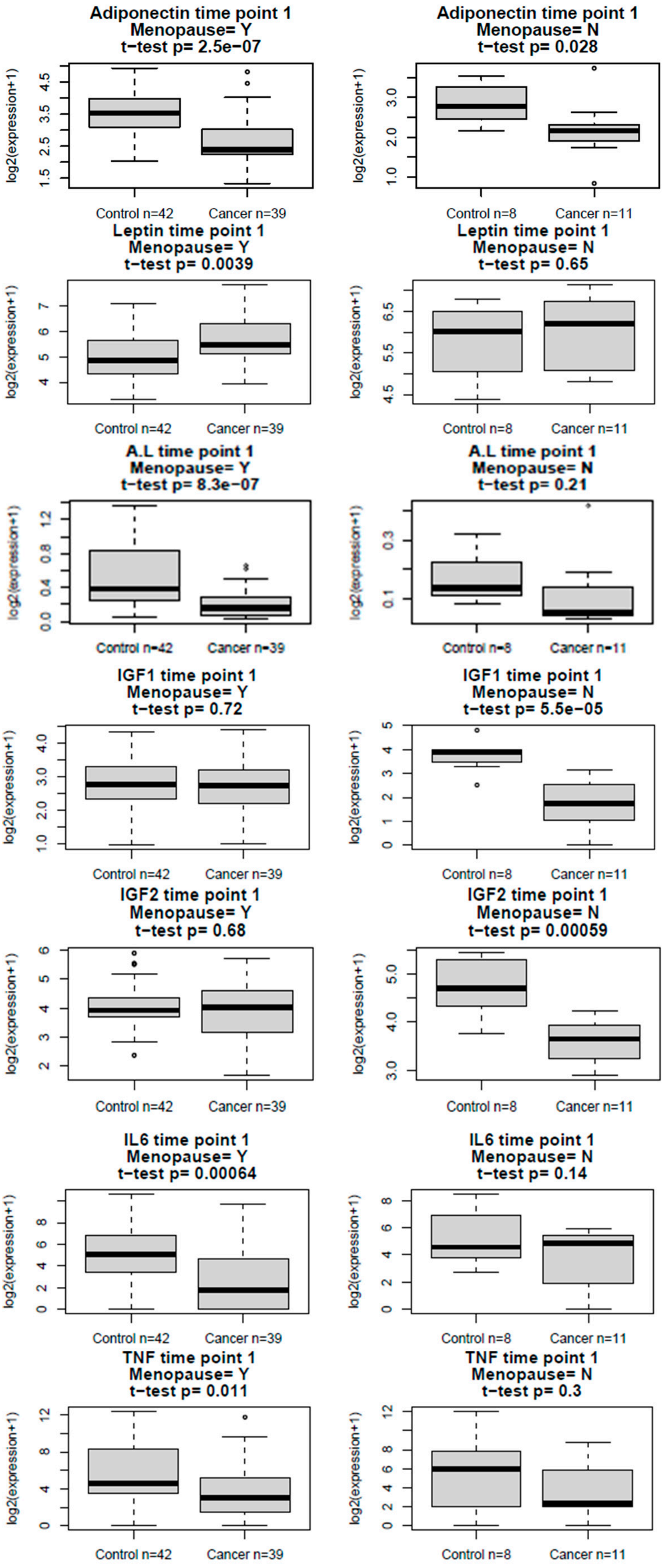

Supplementary Figure S3: Box and whisker plots illustrating the difference in baseline levels of markers between cancer and control populations (subgroup analysis by menopause). $p<0.05$ is significant. Y=yes, N=no.

Difference in levels of markers between cancer and control populations (subgroup analysis by parity)

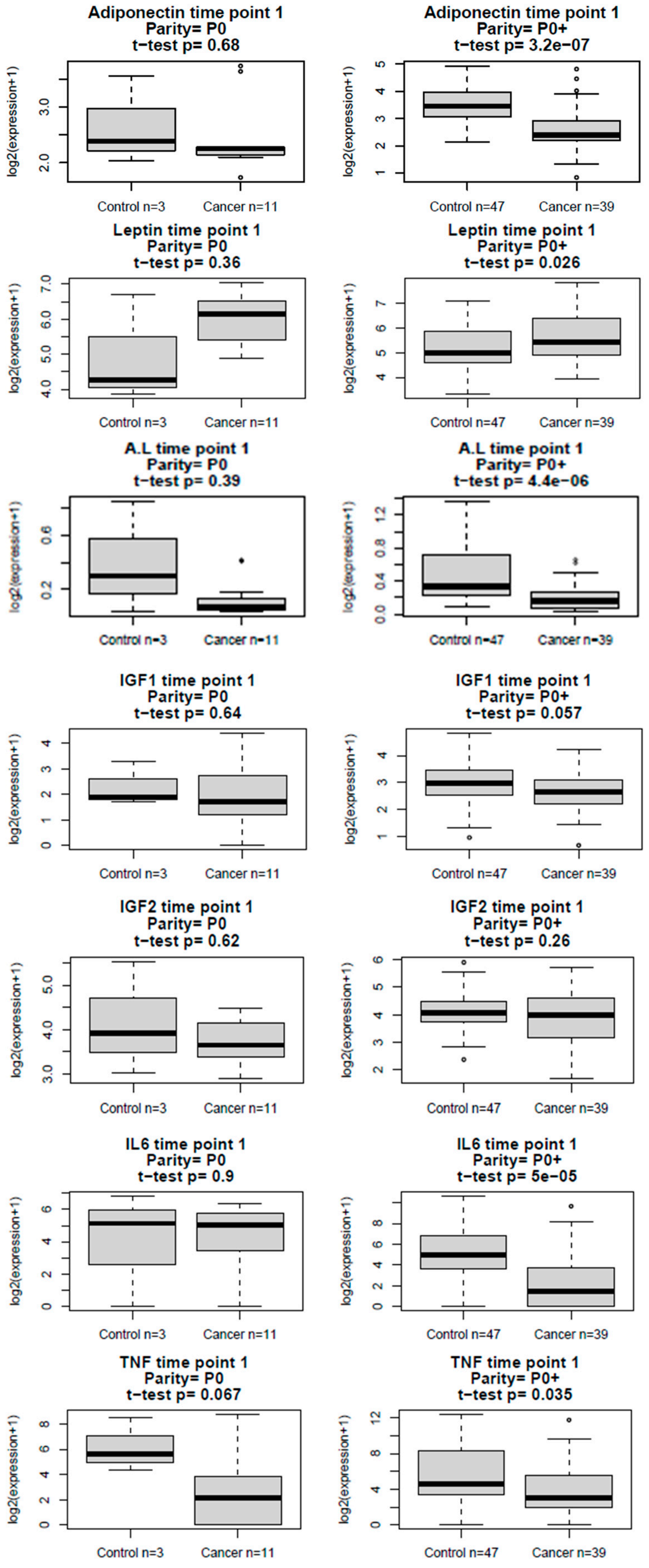

Supplementary Figure S4: Box and whisker plots illustrating the difference in baseline levels of markers between cancer and control populations by (subgroup analysis by parity). p<0.05 is significant. Y=yes, N=no.

Association between levels of biomarkers and demographic characteristics of both the populations:

We examined biomarker levels in connection with the demographic characteristics of both cancer and control patients, which encompassed factors such as BMI, age, ethnicity, parity, HRT and contraception use, diabetes and hypertension. We initiated the analysis with a univariate approach (Supplementary Tables S1 and S2). The significant results in the univariate analyses are represented in Supplementary Fig. S5. We subsequently performed a multivariate analysis on the factors that showed significance in the univariate analyses. The multivariate analyses results are represented in Supplementary Figs. S6 (cancer group) and S7 (control group).

Associations between levels of biomarkers and cancer characteristics in the study population

In our study, we analysed the correlations between the levels of the six markers and several well-recognised prognostic factors of endometrial cancer, including grade, stage, histology, LVSI, MELF, and MSI. Our approach commenced with a preliminary univariate analysis (Supplementary Table S3). The significant results in the univariate analyses are represented in Supplementary Fig. S5. The univariate analysis was thereafter followed by a multivariate analysis of the factors that exhibited significance (Supplementary Fig. S6).

STUDY POPULATION	N (%)	**Adiponectin** (µg/ml) Log Mean (±SE)	P value (adjusted p value)	**Leptin** (ng/ml) Log Mean (±SE)	P value (adjusted p value)	**A/L** Log Mean (±SE)	P value (adjusted p value)	**IL6** (pg/ml) Log Mean (±SE)	P value (adjusted p value)	**TNFα** (pg/ml) Log Mean (±SE)	P value (adjusted p value)	**IGF1** (ng/ml) Log Mean (±SE)	P value (adjusted p value)	**IGF2** (ng/ml) Log Mean (±SE)	P value (adjusted p value)
**BMI**															
18.5-24.9	10 (20)	2.77 (0.26)	0.374	4.66 (0.26)	**0.00006***	0.33 (0.047)	**0.001***	2.41 (0.91)	0.582	4.08 (0.92)	0.500	2.89 (0.30)	0.147	4.29 (0.33)	0.158
≥25.0	40 (80)	2.51 (0.12)	(1.000)	5.9 (0.12)	**(0.0005*)**	0.15 (0.02)	**(0.007*)**	2.96 (0.43)	(1.000)	3.38 (0.43)	(1.000)	2.40 (0.14)	(0.733)	3.76 (0.16)	(0.733)
**Age**															
30-59	17 (34)	2.14 (0.18)	**0.006***	6.06 (0.21)	**0.029***	0.09 (0.03)	**0.001***	3.27 (0.66)	0.453	2.67 (0.66)	0.124	2.17 (0.22)	**0.083**	3.77 (0.25)	0.673
≥60	33 (66)	2.77 (0.13)	**(0.035*)**	5.48 (0.15)	(0.144)	0.23 (0.02)	**(0.009*)**	2.65 (0.47)	(0.906)	3.94 (0.47)	(0.371)	2.65 (0.16)	(0.333)	3.90 (0.18)	(0.906)
**Parity**															
P0	11 (22)	2.43 (0.24)	0.564	5.99 (0.27)	0.203	0.14 (0.05)	0.304	4.28 (0.79)	**0.047***	2.47 (0.82)	0.158	1.99 (0.27)	**0.039***	3.72 (0.30)	0.619
≥P1	39 (78)	2.59 (0.13)	(1.000)	5.59 (0.14)	(0.814)	0.19 (0.03)	(0.912)	2.46 (0.42)	(0.282)	3.80 (0.44)	(0.789)	2.63 (0.14)	**(0.275)**	3.90 (0.16)	(1.000)
**Menopause**															
Yes	39 (78)	2.66 (0.12)	**0.065**	5.59 (0.14)	0.208	0.20 (0.02)	**0.083**	2.64 (0.43)	0.270	3.48 (0.44)	0.893	2.70 (0.13)	**0.002***	3.93 (0.16)	0.356
No	11 (22)	2.17 (0.23)	(0.393)	5.98 (0.27)	(0.830)	0.11 (0.05)	0.413	3.66 (0.81)	(0.830)	3.61 (0.84)	(0.893)	1.74 (0.25)	**(0.011*)**	3.61 (0.30)	0.830
**Diabetes**															
Yes	13 (26)	2.18 (0.21)	**0.045***	5.66 (0.25)	0.933	0.13 (0.04)	0.197	3.25 (0.75)	0.549	4.15 (0.76)	0.334	2.05 (0.25)	**0.047***	3.78 (0.28)	0.744
No	37 (74)	2.68 (0.12)	(0.312)	5.58 (0.15)	1.0001	0.19 (0.03)	0.984	2.72 (0.45)	1.000	3.28 (0.45)	1.000	2.64 (0.15)	0.312	3.89 (0.17)	1.000
**Hypertension**															
Yes	22 (44)	2.49 (0.17)	0.599	5.73 (0.19)	0.714	0.16 (0.03)	0.469	2.23 (0.57)	0.146	3.70 (0.59)	0.660	2.53 (0.19)	0.784	3.92 (0.22)	0.699
No	28 (56)	2.61 (0.15)	(1.000)	5.63 (0.17)	(1.000)	0.19 (0.03)	(1.000)	3.36 (0.50)	(1.000)	3.36 (0.52)	(1.000)	2.46 (0.18)	(1.000)	3.81 (0.19)	(1.000)
**Hormonal contraception**															
Yes	30 (60)	2.44 (0.14)	0.191	5.82 (0.16)	0.162	0.15 (0.03)	0.136	2.63 (0.49)	0.459	3.25 (0.50)	0.425	2.53 (0.17)	0.668	3.87 (0.19)	0.928
No	20 (40)	2.73 (0.17)	(0.972)	5.46 (0.20)	(0.972)	0.22 (0.03)	(0.951)	3.21 (0.61)	(1.000)	3.89 (0.62)	(1.000)	2.42 (0.21)	(1.000)	3.84 (0.23)	(1.000)
**HRT (menopausal, n=39)**															
Yes	10 (26)	2.32 (0.239)	0.103	5.79 (0.29)	0.427	0.13 (0.05)	**0.098**	3.48 (0.88)	0.274	4.14 (0.87)	0.384	2.76 (0.25)	0.774	4.09 (0.35)	0.601
No	29 (74)	2.78 (0.14)	(0.688)	5.52 (0.17)	(1.000)	0.23 (0.03)	(0.688)	2.35 (0.52)	(1.000)	3.25 (0.51)	(1.000)	2.68 (0.15)	(1.000)	3.87 (0.21)	(1.000)

Supplementary Table S1: Levels of the markers with respect to demographic characteristics in the study population. The levels of the markers are expressed in log values that have been used to calculate their significance in the various groups. The p-values are calculated by univariate logistic regression. Significant P-values are marked with asterix (*) and are in bold (p<0.05).

**CONTROL POPULATION**	N (%)	**Adiponectin** (µg/ml) Log Mean (±SE)	P value (adjusted p value)	**Leptin** (ng/ml) Log Mean (±SE)	P value (adjusted p value)	**A/L** Log Mean (±SE)	P value (adjusted p value)	**IL6** (pg/ml) Log Mean (±SE)	P value (adjusted p value)	**TNFα** (pg/ml) Log Mean (±SE)	P value (adjusted p value)	**IGF1** (ng/ml) Log Mean (±SE)	P value (adjusted p value)	**IGF2** (ng/ml) Log Mean (±SE)	P value (adjusted p value)
**BMI**															
18.5-24.9	20 (40)	3.75 (0.14)	**0.004***	4.52 (0.18)	**0.00008***	0.74 (0.06)	**<0.0001***	4.34 (0.61)	0.215	5.06 (0.77)	0.728	3.08 (0.19)	0.276	3.97 (0.17)	0.233
≥25.0	30 (60)	3.23 (0.11)	**(0.025*)**	5.52 (0.15)	**(0.0005*)**	0.29 (0.05)	**(<0.0001*)**	5.32 (0.49)	(0.859)	5.41 (0.63)	(0.859)	2.82 (0.15)	(0.859)	4.24 (0.14)	(0.859)
**Age**															
30-59	17 (34)	3.10 (0.15)	**0.007***	5.32 (0.22)	0.277	0.31 (0.08)	**0.016***	4.43 (0.67)	0.356	4.54 (0.82)	0.282	3.32 (0.19)	**0.014***	4.56 (0.17)	**0.004***
≥60	33 (66)	3.62 (0.11)	**(0.041*)**	5.02 (0.16)	(0.831)	0.55 (0.06)	**(0.069*)**	5.19 (0.48)	(0.831)	5.65 (0.59)	(0.831)	2.72 (0.14)	**(0.069*)**	3.91 (0.13)	**(0.026*)**
**Parity**															
P0	3 (6)	2.66 (0.37)	**0.034***	4.95 (0.55)	0.744	0.39 (0.20)	0.706	3.97 (1.59)	0.538	6.17 (1.98)	0.643	2.30 (0.47)	0.184	4.15 (0.45)	0.963
≥P1	47 (94)	3.49 (0.09)	(0.237)	5.13 (0.14)	(1.000)	0.48 (0.05)	(1.000)	4.99 (0.40)	(1.000)	5.21 (0.50)	(1.000)	2.96 (0.12)	(1.000)	4.13 (0.11)	(1.000)
**Menopause**															
Yes	42 (84)	3.55 (0.09)	**0.004***	4.99 (0.14)	**0.027***	0.53 (0.05)	**0.006***	4.87 (0.43)	0.751	5.24 (0.53)	0.871	2.76 (0.12)	**0.001***	4.02 (0.11)	**0.015***
No	8 (16)	2.84 (0.22)	**(0.024*)**	5.79 (0.32)	**(0.080*)**	0.17 (0.11)	**(0.028*)**	5.22 (0.98)	(1.000)	5.45 (1.22)	(1.000)	3.76 (0.26)	**(0.008*)**	4.73 (0.26)	**(0.060*)**
**Diabetes**															
Yes	3 (6)	3.07 (0.38)	0.321	6.14 (0.53)	**0.053**	0.28 (0.19)	0.321	4.16 (1.59)	0.623	6.49 (1.98)	0.527	1.67 (0.45)	**0.006***	3.57 (0.45)	0.201
No	47 (94)	3.46 (0.09)	(1.000)	5.06 (0.13)	(0.317)	0.48 (0.05)	(1.000)	4.98 (0.40)	(1.000)	5.19 (0.49)	(1.000)	3.00 (0.11)	**(0.039*)**	4.17 (0.11)	(1.000)
**Hypertension**															
Yes	20 (40)	3.39 (0.15)	0.669	5.18 (0.21)	0.724	0.45 (0.08)	0.678	5.46 (0.61)	0.270	4.84 (0.77)	0.467	2.76 (0.18)	0.257	4.01 (0.17)	0.375
No	30 (60)	3.47 (0.12)	(1.000)	5.08 (0.17)	(1.000)	0.49 (0.06)	(1.000)	4.58 (0.49)	(1.000)	5.56 (0.62)	(1.000)	3.03 (0.15)	(1.000)	4.21 (0.14)	(1.000)
**Hormonal contraception**															
Yes	32 (64)	3.49 (0.12)	0.451	5.03 (0.17)	0.371	0.49 (0.06)	0.696	4.76 (0.49)	0.576	4.85 (0.59)	0.248	2.82 (0.15)	0.276	3.98 (0.13)	**0.062**
No	18 (36)	3.35 (0.16)	(1.000)	5.28 (0.22)	(1.000)	0.44 (0.08)	(1.000)	5.22 (0.65)	(1.000)	6.02 (0.79)	(1.000)	3.09 (0.19)	(1.000)	4.40 (0.18)	(0.433)
**HRT(menopausal women,n=42)**															
Yes	20 (48)	3.57 (0.15)	0.263	4.95 (0.21)	0.302	0.52 (0.08)	0.378	4.70 (0.62)	0.639	5.37 (0.77)	0.873	2.82 (0.19)	0.491	3.99 (0.17)	0.294
No	22 (52)	3.35 (0.12)	(1.000)	5.23 (0.17)	(1.000)	0.44 (0.06)	(1.000)	5.08 (0.50)	(1.000)	5.21 (0.63)	(1.000)	2.99 (0.15)	(1.000)	4.23 (0.14)	(1.000)

Supplementary Table S2: Levels of the markers with respect to demographic characteristics in the control population. The levels of the markers are expressed in log values that have been used to calculate their significance in the various groups. The p-values are calculated by univariate logistic regression. Significant P-values are marked with asterix (*) and are in bold (p<0.05).

**STUDY POPULATION**	N (%)	**Adiponectin** (µg/ml) Log Mean (±SE)	P value (adjusted p value)	**Leptin** (ng/ml) Log Mean (±SE)	P value (adjusted p value)	**A/L** Log Mean (±SE)	P value (adjusted p value)	**IL6** (pg/ml) Log Mean (±SE)	P value (adjusted p value)	**TNFα** (pg/ml) Log Mean (±SE)	P value (adjusted p value)	**IGF1** (ng/ml) Log Mean (±SE)	P value (adjusted p value)	**IGF2** (ng/ml) Log Mean (±SE)	P value (adjusted p value)
**Grade**															
1	23 (46)	2.61 (0.16)	0.211	5.89 (0.18)	0.649	0.16 (0.03)	0.694	2.79 (0.59)	0.927	3.59 (0.60)	0.492	2.33 (0.19)	0.139	3.94 (0.19)	0.974
2	13 (26)	2.27 (0.22)	(1.000)	5.75 (0.24)	(1.000)	0.14 (0.04)	(1.000)	2.70 (0.79)	(1.000)	4.29 (0.79)	(1.000)	2.80 (0.25)	(0.975)	3.95 (0.27)	(1.000)
**Stage**															
IA+IB	41 ()	2.55 (0.12)	0.859	5.73 (0.14)	0.423	0.18 (0.02)	0.727	2.93 (0.43)	0.072	3.82 (0.42)	**0.091**	2.40 (0.14)	0.165	3.92 (0.16)	0.364
II+III	9 ()	2.60 (0.26)	(1.000)	5.46 (0.30)	(1.000)	0.19 (0.05)	(1.000)	2.55 (0.91)	(1.000)	2.11 (0.89)	(0.639)	2.88 (0.30)	(0.989)	3.58 (0.33)	(1.000)
**Histology**															
Type 1	36 (72)	2.49 (0.13)	0.328	5.84 (0.15)	**0.040***	0.15 (0.03)	**0.026***	2.76 (0.45)	0.671	3.84 (0.45)	0.169	2.50 (0.16)	0.878	3.94 (0.17)	0.341
Type 2	14 (28)	2.73 (0.21)	(1.000)	5.26 (0.23)	(0.241)	0.26 (0.04)	(0.811)	3.13 (0.73)	(1.000)	2.65 (0.73)	(0.846)	2.46 (0.25)	(1.000)	3.64 (0.27)	(1.000)
**LVSI**															
Yes	15 (30)	2.92 (0.19)	**0.029***	5.61 (0.24)	0.728	0.25 (0.04)	**0.036***	3.06 (0.70)	0.740	3.00 (0.71)	0.396	2.46 (0.24)	0.885	3.66 (0.26)	0.377
No	35 (70)	2.40 (0.13)	(0.205)	5.71 (0.15)	(1.000)	0.15 (0.03)	(0.217)	2.78 (0.46)	(1.000)	3.73 (0.47)	(1.000)	2.50 (0.16)	(1.000)	3.94 (0.17)	(1.000)
**MELF**															
Yes	6 (17)	2.75 (0.31)	0.483	5.06 (0.34)	**0.022***	0.27 (0.06)	**0.056**	4.11 (1.09)	0.178	6.45 (1.13)	**0.015***	2.48 (0.36)	0.692	4.18 (0.38)	0.332
No	29 (83)	2.50 (0.14)	(0.995)	5.94 (0.15)	(0.132)	0.15 (0.03)	(0.281)	2.45 (0.49)	(0.711)	3.26 (0.51)	(0.105)	2.32 (0.16)	(0.996)	3.77 (0.17)	(0.996)
**MSI**															
Yes	10 (24)	2.72 (0.25)	0.428	6.04 (0.29)	0.205	0.16 (0.05)	0.639	2.83 (0.85)	0.931	3.11 (0.87)	0.734	2.33 (0.31)	0.583	3.85 (0.32)	0.972
No	32 (76)	2.49 (0.14)	(1.000)	5.60 (0.17)	(1.000)	0.19 (0.03)	(1.000)	2.91 (0.47)	(1.000)	3.45 (0.49)	(1.000)	2.52 (0.17)	(1.000)	3.87 (0.18)	(1.000)

Supplementary Table S3: Levels of the markers with respect to endometrial cancer characteristics. The levels of the markers are expressed in their respective units as measured by ELISA in plasma of cancer patients. The p-values are calculated by univariate logistic regression. * Significant P-value (<0.05).

Univariate Analyses

STUDY PATIENTS

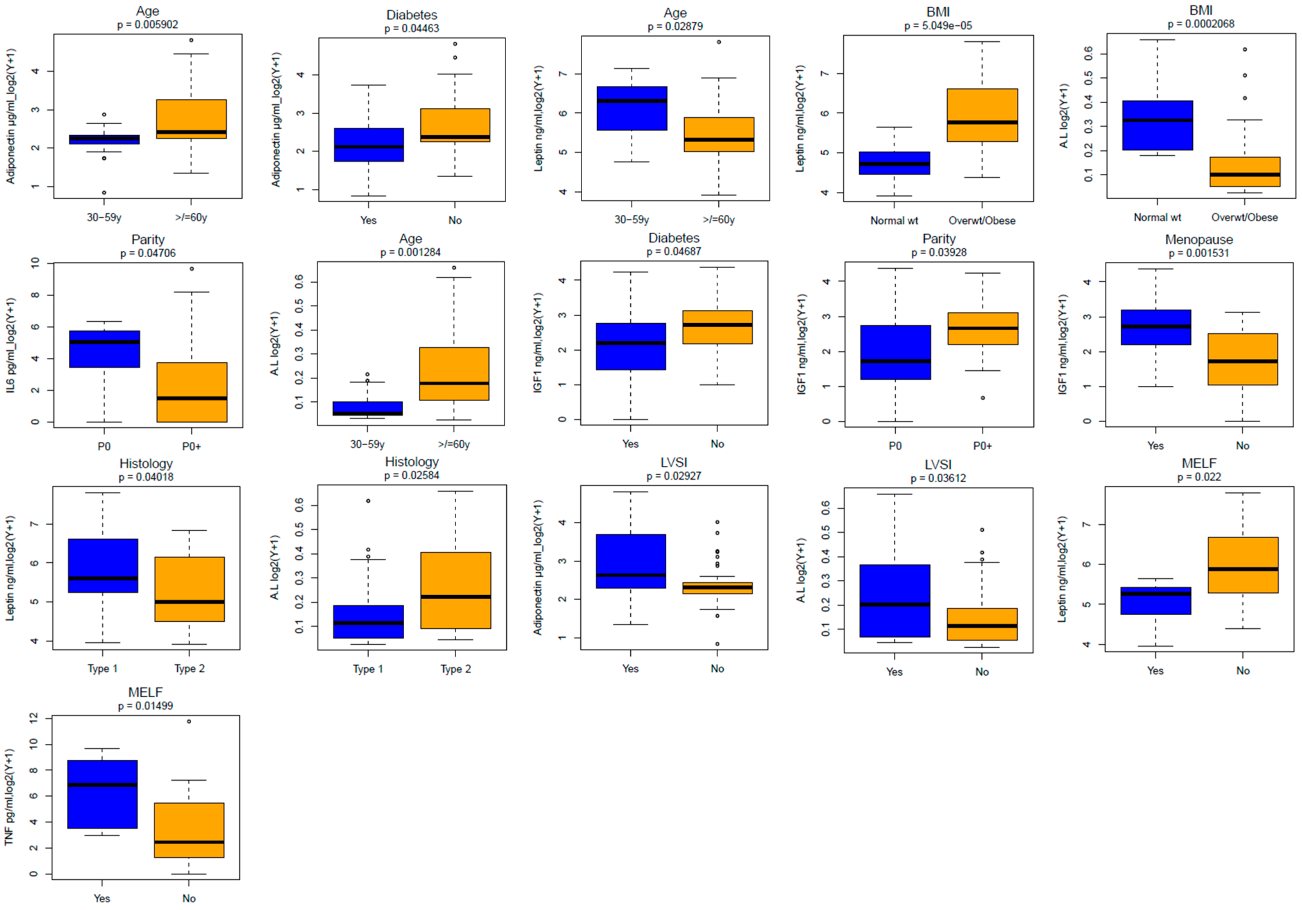

CONTROL PATIENTS

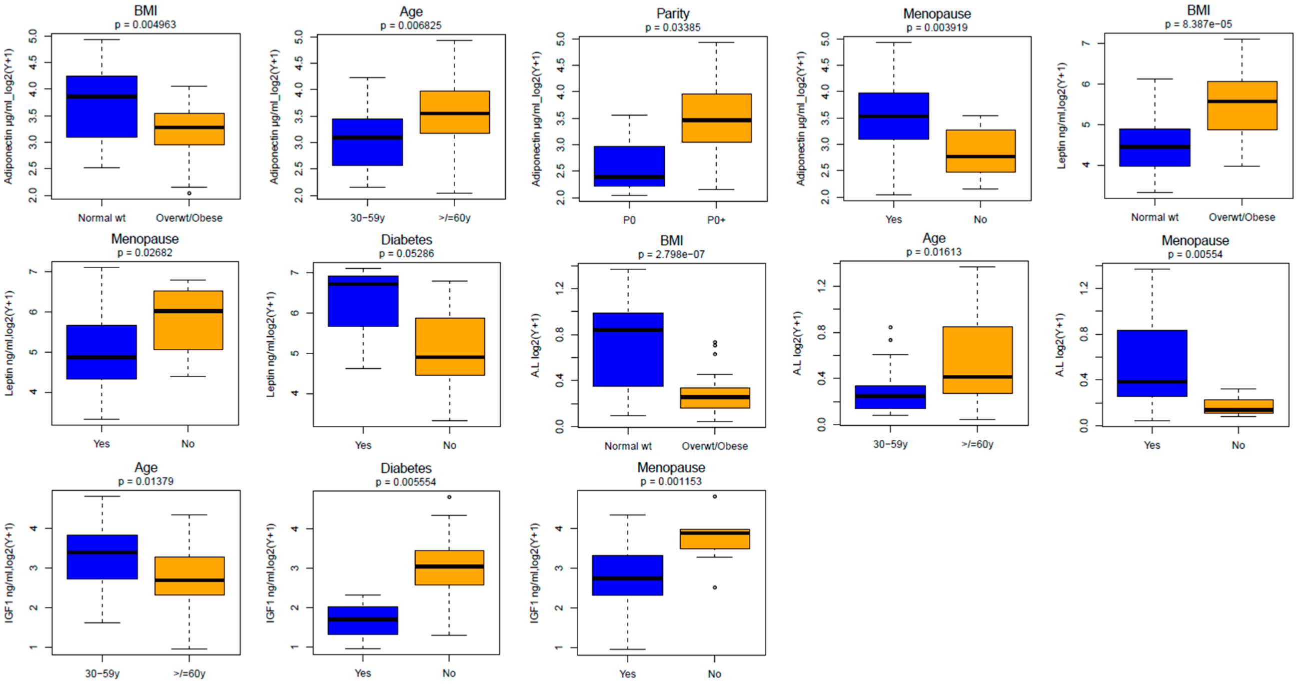

Supplementary Figure S5: Box and whisker plots demonstrating the association (univariate linear regression) between prognostic factors and the biomarkers in study and cancer patients. X axis- prognostic factors groups**;** Y axis- log2 [(level of the marker) +1]. The 25th and 75th percentiles are represented by the lower and upper boundaries of the rectangles, respectively, while the median is indicated by the horizontal line inside the rectangles. The whiskers extend from the box

denoting the minimum and maximum values. The blue and orange colours are only to differentiate between the different categories within each variable.

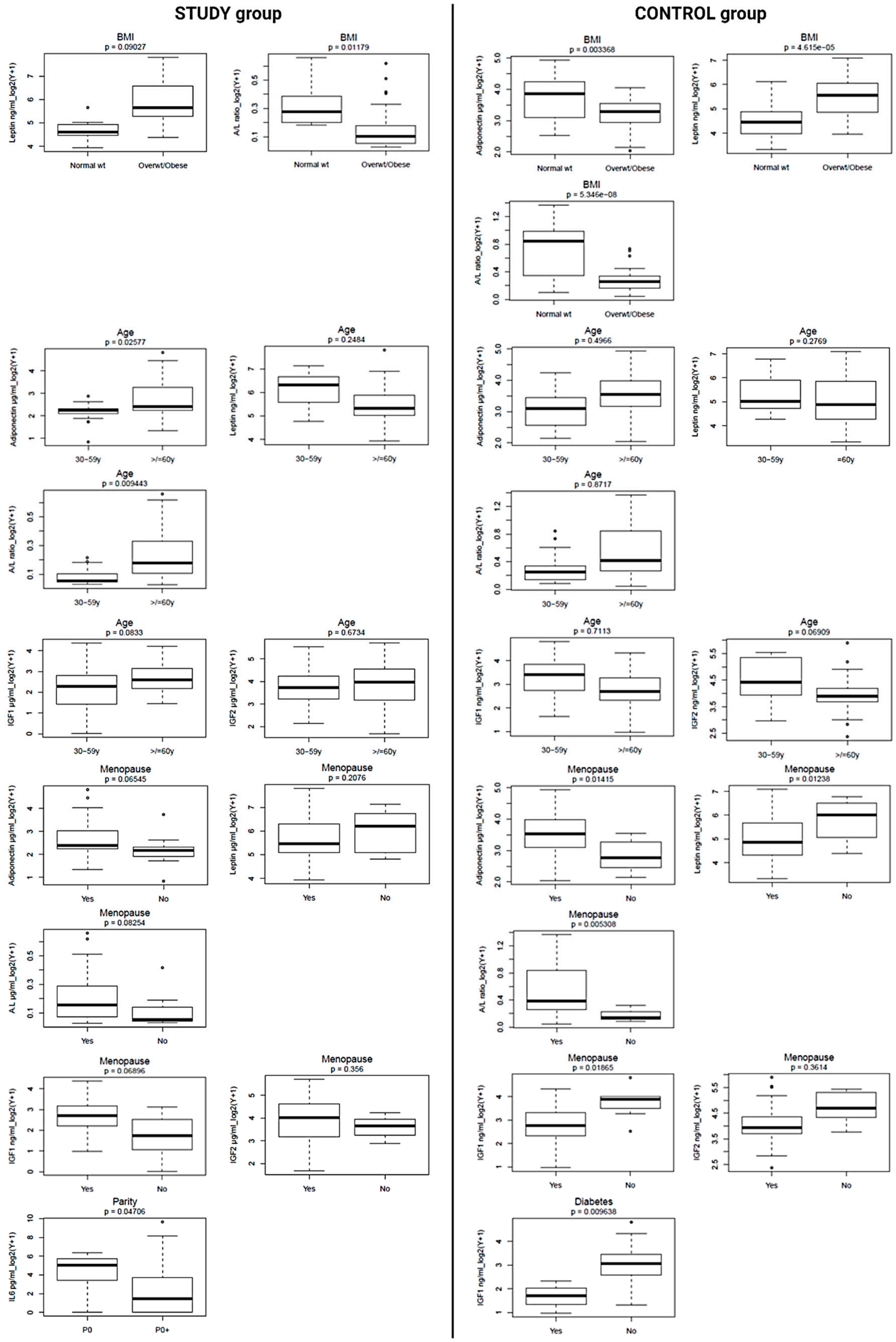

Multivariate Analyses

Supplementary Figure S6: Box and whisker plots demonstrating the associations between patient demographic factors and the biomarkers in study and control patients using multivariate linear regression (total n=50, sample size for each subgroup have been mentioned in table 1). X axis- demographic factors' groups; Y axis- log2 [(level of the marker) +1]. The 25th and 75th percentiles are represented by the lower and upper boundaries of the rectangles, respectively, while the median is indicated by the horizontal line inside the rectangles. The whiskers extend from the box denoting the minimum and maximum values.

STUDY group

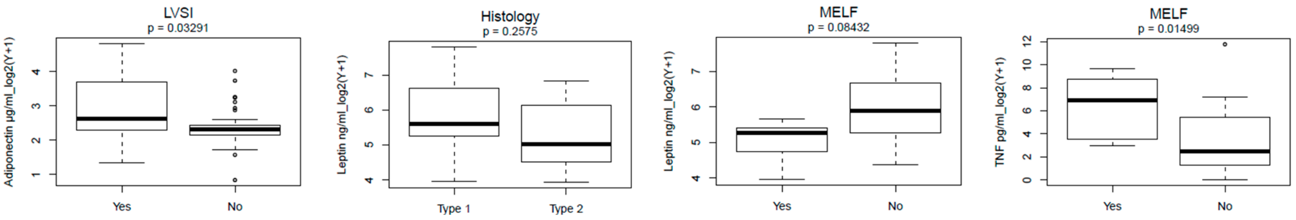

Supplementary Figure S7: Box and whisker plots demonstrating the associations between endometrial cancer characteristics and the biomarkers in study population using multivariate linear regression (total n=50, sample size for each subgroup have been mentioned in table 1). X axis- endometrial cancer characteristics' groups; Y axis- log2 [(level of the marker) +1]. The 25th and 75th percentiles are represented by the lower and upper boundaries of the rectangles, respectively, while the median is indicated by the horizontal line inside the rectangles. The whiskers extend from the box denoting the minimum and maximum values.

Correlation between the markers

The interactions between various adipocytokines and the IGF systems are complex, and not yet fully elucidated. There is a growing body of evidence indicating adipocytokines and IGFs are physiologically linked and work synergistically to modulate cancer risk via interconnected pathways. With the aim of investigating these relationships, we assessed how the levels of these markers were correlated in cancer and control populations. For this, the Pearson's r value was calculated between the markers in cancer and control populations (Supplementary Figure S8).

In both populations, moderate correlation was noted between IGFs 1 and 2 levels (cancer: r=0.51, p=0.0001; adjusted p=0.004; control: r=0.64, p<0.0001; adjusted p<0.0001) and between IL6 and TNFα levels (cancer: r =0.48, p=0.0005, adjusted p=0.013 ; control: r=0.63, p<0.0001, adjusted p=0.0002). A low inverse correlation was seen between IGF1 and leptin (r=-0.30, p=0.037, adjusted p=0.962) in the cancer population. This indicated that as IGF1 level decreased, leptin level tended to increase in the cancer population. However, no such inverse

correlation was noted in the control population. Leptin shows low positive significant correlations with both IL6 and TNFα in the control population (r=0.36, p=0.009, adjusted p=0.258 ; and r=0.32, p=0.023, adjusted p=0.549, respectively) which are not present in the cancer population. This indicates there are distinct patterns of correlations between biomarkers in the cancer population compared to the control population, potentially highlighting unique biological interactions in the context of cancer development or progression, that is different

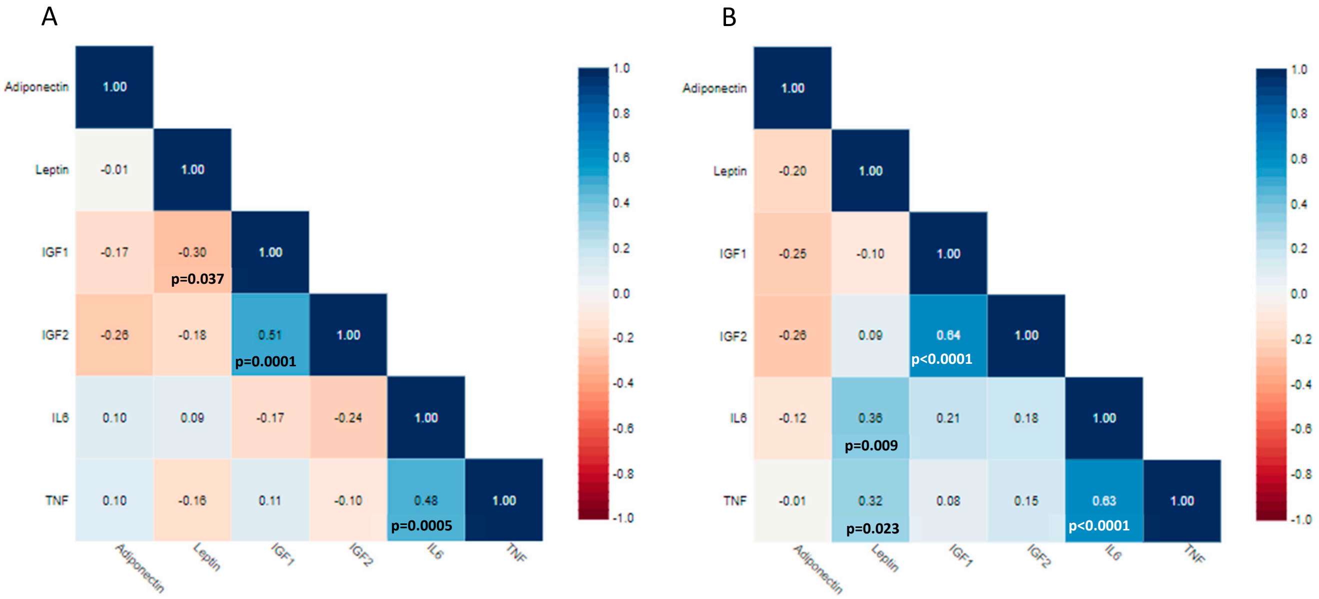

from non-cancer population.

Supplementary Figure S8: Association between all 6 markers. A) Cancer population – at baseline B) Control population. Red to blue end of the scale illustrates strong negative (Pearson's r=-1) to strong positive (Pearson's r=+1) correlation with white indicating no correlation Pearson's (r=0). Significant p-values (<0.05) are mentioned in the figure.

Pearson's correlations

Pearson's r value was calculated to assess correlation between the markers in cancer and control populations. p-value of <0.05 indicated that a correlation calculation was significant and not purely by chance.

Pearson r-value	Interpretation
r=1 or, r=-1	Perfect positive/ negative linear correlation
1>r≥0.8 or, −0.8>r>−1	Strong positive/ negative linear correlation
0.8>r≥0.4 or, −0.4>r≥−0.8	Moderate positive/ negative linear correlation
0.4>r>0 or, 0>r≥−0.4	Weak positive/ negative linear correlation
r=0	No correlation
p<0.05	Correlation calculated is significant

Supplementary Table S4: Demonstrating the interpretation of Pearson's r value

Some additional information on endometrial cancer

Risk factors for endometrial cancer:

The biomarker levels were correlated with different demographic characteristics of the cancer and control patients such as BMI, age, ethnicity, parity, use of HRT/contraception, diabetes, hypertension, past history of cancer and family history. We planned to seek these specific correlations as some of these demographic factors are associated with increased risk of developing endometrial cancer such as age (>60 years), nulliparity (a woman who has never given birth), obesity, menopause, oestrogen only hormone replacement therapy, diabetes, hypertension, past history of cancer (granulosa cell tumour of ovary), use of tamoxifen for treatment of breast cancer, family history of endometrial-bowel cancer (Lynch syndrome) and others are associated with reduced risk of endometrial cancer such as use of combined oral contraceptive pill, Mirena intrauterine contraceptive device, and nulliparity.

Most endometrial cancer patients fall within the 75-79 years age bracket[1].

Parity, or the number of children a woman has, impacts endometrial cancer risk. Parous women have a lower risk compared to nulliparous women, and a meta-analysis by Wu et al. revealed an inverse relationship between increasing parity and endometrial cancer risk [2, 3].

Obesity is linked to endometrial cancer, with 34% of cases attributed to it [4].

Smoking's correlation with endometrial cancer is controversial; Zhou et al.'s meta-analysis indicated reduced risk among ever-smokers [5].

Endometrial cancer is often oestrogen-related, and unopposed oestrogen exposure, especially after menopause or with oestrogen-only hormone replacement therapy (HRT), increases risk [6].

Postmenopausal women are at the highest risk due to oestrogen from adipose tissue becoming the primary source after ovarian oestrogen production stops [7].

Oral contraceptives lower risk by 26-43% after 5+ years of use, with protection lasting up to 20 years post-use [1]. The Mirena intrauterine device, releasing progesterone, reduces endometrial cancer risk by 19% due to endometrial thinning.

Oestrogen-only HRT elevates risk, while continuous combined HRT (oestrogen and progesterone together daily) lowers it by 22% [8].

Diabetes and hypertension are established risk factors. Diabetics face a 40-81% higher risk [19]. Hypertension is associated with an increased risk, with a relative risk of 1.61 according to a meta-analysis by Aune et. al. [10].

Regarding the medical history of cancer, it's important to consider the use of Tamoxifen, a medication employed in breast cancer treatment. Tamoxifen is associated with endometrial lining thickening and heightened endometrial cancer risk [6]. In terms of family medical history, a significant factor to note is a history of endometrial cancer, potentially with concurrent colon cancer, within the family. This condition is referred to as hereditary nonpolyposis colon cancer (HNPCC) or Lynch syndrome [6].

Correlating biomarkers with patient demographics can aid in tailoring personalized treatment plans based on individual characteristics, potentially improving treatment outcomes. We compared the levels of the markers in with the demographic characteristics of both study and control populations to investigate how the biomarker are altered in cancer populations.

FIGO staging of Endometrial cancer – old and new

Endometrial cancer is characterised by its grade, stage, and histology. The staging of endometrial cancer used in the study is based on the International Federation of Gynecology and Obstetrics (FIGO) 2009 classification[11] (Supplementary Table S5). However, since the commencement of the study in 2021, a new staging has been proposed by FIGO in 2023 [12] which is presented in Supplementary Table S6, although it has not been able to re-stage the cases using the new staging.

Stages	Description
I	Cancer confined to body of uterus
1A	Cancer limited to endometrium or, involving <50% of myometrium
1B	Cancer invasion into ≥ 50% of myometrium
II	Cancer spread to cervical stroma, but not beyond uterus
III	Cancer spread beyond uterus but confined to pelvis
IIIA	Cancer spread to outer surface of the uterus and/or the ovaries and fallopian tubes
IIIB	Cancer spread into the vagina and/or into parametrium (tissues around the uterus)
IIIC	Cancer spread to pelvic and/or para-aortic lymph nodes
IV	Cancer spread to other organs
IVA	Cancer spread to bladder and/or bowel
IVB	Distant metastasis, including intra-abdominal metastasis and /or inguinal lymph nodes

Supplementary Table S5. FIGO 2009 staging of endometrial cancer (used in the study) [11]

Stages	Description
I	Cancer confined to uterine corpus and ovary
1A	Disease limited to the endometrium OR non- aggressive histological type, i.e. low- grade endometroid, with invasion of less than half of myometrium with no or focal lymphovascular space involvement (LVSI) OR good prognosis disease
	1A1 Non- aggressive histological type limited to an endometrial polyp OR confined to the endometrium
	1A2 Non- aggressive histological types involving less than half of the myometrium with no or focal LVSI
	1A3 Low- grade endometrioid carcinomas limited to the uterus and ovary
1B	Non- aggressive histological types with invasion of half or more of the myometrium, and with no or focal LVSI
1C	Aggressive histological types limited to a polyp or confined to the endometrium
II	Invasion of cervical stroma without extrauterine extension OR with substantial LVSI OR aggressive histological types with myometrial invasion
IIA	Invasion of the cervical stroma of non- aggressive histological types
IIB	Substantial LVSI of non- aggressive histological types
IIC	Aggressive histological types with any myometrial involvement
III	Local and/or regional spread of the tumour of any histological subtype
IIIA	Invasion of uterine serosa, adnexa, or both by direct extension or metastasis
	IIIA1 Spread to ovary or fallopian tube (except when meeting stage IA3 criteria)
	IIIA2 Involvement of uterine subserosa or spread through the uterine serosa
IIIB	Metastasis or direct spread to the vagina and/or to the parametria or pelvic peritoneum
	IIIB1 Metastasis or direct spread to the vagina and/or the parametria
	IIIB2 Metastasis to the pelvic peritoneum
IIIC	Metastasis to the pelvic or para- aortic lymph nodes or both
	IIIC1 Metastasis to the pelvic lymph nodes
	IIIC1i Micrometastasis
	IIIC1ii Macrometastasis
	IIIC2 Metastasis to para- aortic lymph nodes up to the renal vessels, with or without metastasis to the pelvic lymph nodes
	IIIC2i Micrometastasis
	IIIC2ii Macrometastasis
IV	Spread to the bladder mucosa and/or intestinal mucosa and/or distance metastasis
IVA	Invasion of the bladder mucosa and/or the intestinal/bowel mucosa
IVB	Abdominal peritoneal metastasis beyond the pelvis
IVC	Distant metastasis, including metastasis to any extra- or intra- abdominal lymph nodes above the renal vessels, lungs, liver, brain, or bone

Supplementary Table S6. FIGO 2023 staging of endometrial cancer. Macrometastases are >2 mm in size, micrometastases are 0.2– 2 mm and/or >200 cells, and isolated tumour cells are ≥0.2 mm and ≤200 cells [12].

References

1. Cancer Research UK. Uterine cancer incidence statistics. http://www.cancerresearchuk.org/health-professional/cancer-statistics/statistics-by-cancer-type/uterine-cancer/incidence#heading-One.

2. Wu, Q.-J. *et al.* Parity and endometrial cancer risk: a meta-analysis of epidemiological studies OPEN. *Nature Publishing Group* **5**, 14243 (2015).

3. Dossus, L. *et al.* Reproductive risk factors and endometrial cancer: the European Prospective Investigation into Cancer and Nutrition. *Int J Cancer* **127**, 442–451 (2010).

4. Brown, K. F. *et al.* The fraction of cancer attributable to modifiable risk factors in England, Wales, Scotland, Northern Ireland, and the United Kingdom in 2015. *Br J Cancer* **118**, (2018).

5. Zhou, B. *et al.* Cigarette smoking and the risk of endometrial cancer: a meta-analysis. *Am J Med* **121**, (2008).

6. American Society of Clinical Oncology. Uterine Cancer: Risk Factors and Prevention. 09/2022. . https://www.cancer.net/cancer-types/uterine-cancer/risk-factors-and-prevention.

7. The American Cancer Society medical and editorial content team. Endometrial Cancer Risk Factors. *American Cancer Society* (2019).

8. Brinton, L. A. & Felix, A. S. Menopausal hormone therapy and risk of endometrial cancer. *Journal of Steroid Biochemistry and Molecular Biology* **142**, (2014).

9. Zhang, Z. H., Su, P. Y., Hao, J. H. & Sun, Y. H. The role of preexisting diabetes mellitus on incidence and mortality of endometrial cancer: A meta-analysis of prospective cohort studies. *International Journal of Gynecological Cancer* **23**, (2013).

10. Aune, D., Sen, A. & Vatten, L. J. Hypertension and the risk of endometrial cancer: A systematic review and meta-analysis of case-control and cohort studies. *Sci Rep* **7**, (2017).

11. Pecorelli, S. Revised FIGO staging for carcinoma of the vulva, cervix, and endometrium. *Int J Gynaecol Obstet* **105**, 103–104 (2009).

12. Berek, J. S. *et al.* FIGO staging of endometrial cancer: 2023. *International Journal of Gynecology & Obstetrics* **162**, 383–394 (2023).
